# OTUB2 induces M2 tumor-associated macrophage polarization and increases CD274 expression in gastric cancer cells to aggravate the progression of gastric cancer

**DOI:** 10.1038/s41419-026-08743-9

**Published:** 2026-04-15

**Authors:** Jie Li, Juan Sun, Chenggang Zhang, Yixuan He, Zicheng Zheng, Guanmo Liu, Yuqin Liu, Weiming Kang, Xin Ye

**Affiliations:** 1 Department of General Surgery, Peking Union Medical College Hospital, Chinese Academy of Medical Sciences & Peking Union Medical College, Beijing, People’s Republic of China; 2 Department of Breast Surgery, Peking Union Medical College Hospital, Chinese Academy of Medical Sciences & Peking Union Medical College, Beijing, People’s Republic of China; 3 Department of Anesthesiology, Peking Union Medical College Hospital, Chinese Academy of Medical Sciences & Peking Union Medical College, Beijing, People’s Republic of China; 4 Department of Pathology, Cell Resource Center, Institute of Basic Medical Sciences & School of Basic Medicine, Chinese Academy of Medical Sciences & Peking Union Medical College, Beijing, People’s Republic of China

**Keywords:** Gastric cancer, Immune evasion, Ubiquitylation

## Abstract

Gastric cancer (GC) is often diagnosed at an advanced stage due to the absence of early symptoms and low screening rates, resulting in poor prognosis. The progression of GC is closely related to the immune response within the tumor microenvironment (TME). Tumor-associated macrophages (TAMs), particularly the M2 subtype, are the most prevalent inflammatory cells in the TME and play a crucial role in GC. Tumor cells also evade immune surveillance by upregulating CD274. OTUB2, a deubiquitinase, regulates tumor progression by deubiquitinating substrate proteins. However, the role of OTUB2 in TAMs polarization and immune evasion in GC remains unclear. Stable OTUB2 overexpression and knockdown cells were cocultured with M0 macrophages to study TAMs polarization. Flow cytometry was used to analyze M2 TAMs and CD274 expression on GC cells. Cytokine secretion was evaluated by ELISA. T cell killing assays were performed by co-culturing GC cells with CD8^+^ T cells. Co-immunoprecipitation and Western blotting assessed the ubiquitination levels of YAP, TAZ and CD274. In vivo studies were conducted to evaluate OTUB2’s effect on TAMs polarization, immune evasion and GC progression. Immunohistochemistry of GC tissues was performed to investigate the correlation between OTUB2 and TME components. OTUB2 overexpression activated YAP/TAZ to increase TGF-β1 and M2 TAMs polarization by inhibiting SMAD7. It also enhanced CD274 expression, promoting immune evasion. OTUB2 deubiquitinated YAP, TAZ, and CD274, preventing their degradation. In vivo, OTUB2 increased M2 TAMs polarization and CD274 expression, exacerbating GC progression. Immunohistochemistry confirmed a positive correlation between OTUB2, M2 TAMs infiltration and CD274 levels and a negative correlation with CD8^+^ T cell infiltration. Kaplan–Meier analysis showed reduced overall survival in GC patients with high OTUB2 expression. OTUB2 promotes M2 TAMs polarization and immune evasion in GC. Targeting OTUB2 offers a promising strategy to reshape the GC TME and improve the efficacy of immune checkpoint inhibitors.

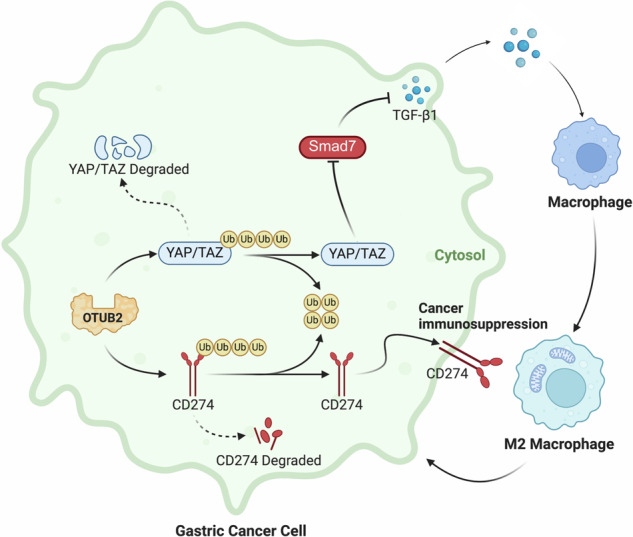

## Introduction

Gastric cancer (GC) ranks as one of the most widespread malignant digestive tumors worldwide, ranking fifth in both incidence and mortality according to the 2022 Global Cancer Statistics [1]. Despite advancements in treatment modalities, including neoadjuvant therapy, surgical intervention, adjuvant chemotherapy, targeted therapy, and immunotherapy, the overall prognosis for patients with GC remains dismal [2]. While comprehensive treatment approaches have led to improvements in the 5-year survival rate [3], GC continues to pose a significant public health challenge in China, driven by dietary patterns, *Helicobacter pylori* infection, and limited screening uptake [4, 5]. Early-stage GC is often asymptomatic, leading to more than 80% of patients being diagnosed at a locally advanced or metastatic stage [6]. Although immune checkpoint inhibitors (ICIs) have shown promise in the management of advanced cancers [7], their efficacy in patients with GC is limited, largely due to the absence of effective molecular subtype-specific strategies [8, 9]. Elucidating the molecular mechanisms underlying GC progression and immune evasion is therefore critical for identifying promising therapeutic targets and enhancing the therapeutic benefit of ICIs.

The ubiquitin‒proteasome system (UPS) is a critical regulatory mechanism in eukaryotic cells that plays a pivotal role in fundamental processes such as cell proliferation, cell differentiation and signal transduction [10]. Intracellular protein ubiquitination–deubiquitylation is a comprehensive, dynamic and highly specific biological process that not only regulates many biological processes, including protein sorting, degradation, DNA repair, transcriptional activation and gene silencing, but is also a hot spot for clinical translational therapy [11, 12]. OTUB2 (OTU deubiquitinase, ubiquitin aldehyde binding 2) is part of the OTU family of deubiquitinating enzymes, which is specific for identifying different types of ubiquitin chains and plays a critical role in regulating signaling cascade pathways by deubiquitinating substrate proteins [13]. Recently, studies have reported that OTUB2 plays a significant role in tumor progression. Zhang et al. demonstrated that OTUB2 promotes breast cancer (BC) metastasis, with higher OTUB2 expression correlating with poorer patient survival, identifying it as a potential therapeutic target in BC [14]. Additionally, OTUB2 has been demonstrated to increase tumorigenesis in non-small cell lung cancer by stabilizing U2AF2, which in turn activates the Akt/mTOR pathway and promotes the Warburg effect [15]. Our previous work revealed that OTUB2 drives GC progression by activating the AKT pathway through deubiquitination of KRT80 [16]. However, the role of OTUB2 in GC remains incompletely understood, particularly with respect to its influence on the tumor microenvironment (TME) and immune evasion. Further investigation into the mechanisms by which OTUB2 modulates the TME and facilitates immune escape in GC will provide valuable understanding of the broader role of ubiquitination–deubiquitination in GC pathogenesis.

The progression and prognosis of GC are not only related to the characteristics of the tumor itself but also closely related to the TME and immune response [17, 18]. Tumor-associated macrophages (TAMs), essential players in innate immunity, have pivotal functions within the TME. TAMs are usually classified into M1 types (classically activated) and M2 types (alternatively activated). M1 TAMs exhibit antitumor properties, whereas M2 TAMs contribute to tumor progression by secreting immunosuppressive cytokines, including IL-10 and TGF-β [19]. Increasing evidence suggests that TAMs, as active infiltrators in the TME, significantly contribute to tumor cell proliferation, invasion, migration, chemoresistance, angiogenesis, and immune regulation in GC [20]. Immune evasion, a hallmark of cancer, plays a critical role in GC progression [18]. Studies have demonstrated that tumor cells evade immune clearance by upregulating CD274 (programmed death ligand 1, PD-L1) and binding to CD279 (programmed death receptor 1, PD-1) on T cells, thereby suppressing T-cell proliferation and function and weakening the antitumor immune response [21]. Immune checkpoint inhibitors (ICIs), which disrupt the PD-L1/PD-1 interaction, increase host immune-mediated tumor cell clearance. However, the relationships among the ubiquitination–deubiquitination processes in GC, TAMs polarization within the TME and immune evasion by GC cells remain insufficiently understood.

In this study, we demonstrated that OTUB2, a deubiquitinase, promotes YAP/TAZ expression by deubiquitinating these proteins, leading to increased secretion of TGF-β1 via repressing SMAD7 by GC cells. This, in turn, drives the polarization of M2 TAMs within the TME, which further promotes the malignant behaviors of GC cells. Additionally, we found that OTUB2 directly deubiquitinates CD274, thereby upregulating CD274 expression on the surface of GC cells and facilitating immune evasion by inhibiting CD8^+^ T-cell-mediated clearance. Immunohistochemical (IHC) analysis revealed that OTUB2 is markedly expressed in tumor tissues and is positively associated with YAP/TAZ, TGF-β1, CD274 and the infiltration of M2 TAMs (marked by CD163 and CD206), while inversely related to CD8^+^ T-cell infiltration (marked by CD3 and CD8) in the GC TME. Kaplan–Meier survival analysis further indicated that elevated OTUB2 expression is a significantly linked to reduced overall survival in GC patients. These results suggest that OTUB2 may serve as a promising candidate for reshaping the TME and improving the efficacy of ICIs in GC therapy.

## Materials and methods

### Tissue specimens

#### Tissue microarray samples

Sixty-three pairs of GC tissues and corresponding adjacent non-cancerous tissues were collected from GC patients who underwent radical gastrectomy. After the intraoperative specimens were resected, appropriate amounts of tumor tissues and adjacent tissues were taken without affecting the pathological diagnosis, immediately fixed in formalin and embedded in paraffin within 1 week.

#### GC samples with prognostic information

Clinicopathological data from 178 patients who underwent radical gastrectomy for GC and received a pathological diagnosis at the Department of General Surgery, PUMCH, CAMS, between 2015 and 2017 were retrospectively collected. Patient survival status was obtained through telephone follow-up, and survival time was recorded. The surgically obtained tissue blocks preserved through formalin fixation and paraffin embedding (FFPE) were retrieved from the Department of Pathology and sectioned for IHC staining in subsequent analyses.

The study was approved by the Ethics Committee and Institutional Review Board, PUMCH, CAMS (Approval No. K1447). In accordance with the guidelines set out in the Declaration of Helsinki, the written informed consent of each patient or his/her relative was obtained for the collection of specimens.

### Cell culture and induction of M0 macrophages and M2 TAMs

The cell lines (AGS (RRID: CVCL_0139), MKN45 (RRID: CVCL_0434), HGC27 (RRID: CVCL_1279), HEK-293T (RRID: CVCL_0063), MFC, human mononuclear cells (THP-1, RRID: CVCL_0006) and PUMC-HGC1) used in this study were obtained from the Cell Resource Center, Institute of Basic Medical Sciences, CAMS. Phorbol myristate acetate (100 ng/ml) was introduced into the THP-1 cells for induction. After 24 h, the adherent cells were monocyte-derived M0 macrophages. IL-4 (20 ng/ml) and IL-13 (20 ng/ml) were subsequently applied to M0 macrophages for 3 days to induce M2 TAMs.

### Noncontact coculture of tumor cells and macrophages

M0 macrophages were initially seeded into a six-well plate. A culture chamber with a 0.4-μm pore size was then positioned on the same plate, followed by the addition of GC cells into the chamber for noncontact coculture. Similarly, GC cells were plated in a separate six-well plate, and a culture chamber with a 0.4-μm pore size was placed on the same plate. M2 TAMs were then introduced into the chamber for noncontact coculture.

### Overexpressed and knocked down OTUB2 in GC cells

The upregulated and downregulated lentiviruses of OTUB2 used in this study were constructed by Gene Co., Ltd. (Shanghai, China). HGC27 was selected to knockdown OTUB2 at a multiplicity of infection (MOI) of 20, whereas AGS was chosen to overexpress OTUB2 at MOI of 30. Puromycin (Beyotime, China) was subsequently used at a concentration of 5 μg/ml to screen stably transfected upregulated and downregulated GC cells, and the results were verified via western blotting. The sequences of shOTUB2 are shown in Supporting Information Table S1.

### Cell viability assay

The assessment of cell proliferation was conducted via the Cell Counting Kit-8 (CCK-8) and colony formation assays. For the CCK-8 assay, 800 cells were plated into each well of a 96-well plate with 200 μl of medium. The CCK-8 solution was diluted with cell culture medium at a 1:9 ratio under dark conditions, and 100 μl of this mixture was added to each well. The absorbance was determined at 450 nm to assess cell viability. For the colony formation assay, 800 GC cells were plated in each well of a six-well plate with 3 ml of medium. After approximately 8 days of culture, the colonies were fixed with 4% formaldehyde and stained with crystal violet.

### Flow cytometry

AGS cells were cultured at a density of 300,000 cells per well, while HGC27 cells were cultured at 200,000 cells per well in a six-well plate. After 24 h, the cells were collected and fixed for at least one day by sequentially adding 1 ml of PBS, followed by three additions of 1 ml of absolute ethanol. The cells were then filtered through 40-μm filter membranes and transferred to flow cytometry tubes. An appropriate volume of periodic detection reagent (KGA9101, KeyGEN BioTECH, China) was added under dark conditions, and the cell cycle phase was analyzed via flow cytometry (LSRFortessa, Becton, Dickinson and Company, USA). Under the same conditions, AGS and HGC27 cells were plated in a six-well plate. Following a 24-h period, 4 μmol of hydrogen peroxide was added to each well, and the mixture was incubated for 1 h to induce apoptosis. The cells were then collected via EDTA-free trypsin, including the cell supernatant. Each sample was mixed with 0.5 ml of Annexin V binding buffer, filtered through a 40-μm filter membrane, and transferred to flow cytometry tubes. APC-annexin V (5 μl) and PI (5 μl) (KGA1107, KeyGEN BioTECH, China) were added under dark conditions, and the percentage of apoptotic cells was determined via flow cytometry.

After noncontact coculture with GC cells, the macrophages were filtered through a 40 μm filter membrane. Subsequently, 5 μl of FITC-conjugated anti-human CD206 (321104, Biolegend, USA) and 5 μl of PE-conjugated anti-human CD163 (326506, Biolegend, USA) were added under dark conditions. The ratio of M2 TAMs was quantified via flow cytometry.

GC cells were harvested, subjected to filtration through a 40 μm filter membrane, and stained with 5 μl of APC-conjugated anti-human CD274 (B7-H1, PD-L1) antibody (Thermo Fisher Scientific Cat# 17-5983-42, RRID: AB_10597586) in the dark. CD274 expression in GC cells was directly measured via flow cytometry. For indirect detection of CD274 expression, GC cells were filtered through a 40 μm filter membrane and incubated with 5 μl of biotinylated recombinant PD-1-Fc chimera (789404, Biolegend, USA) for 2 h. After washing, 5 μl of Brilliant Violet 421™ Streptavidin (405226, Biolegend, USA) was added under dark conditions, and CD274 expression was analyzed via flow cytometry.

### T-cell killing assay

First, CD8^+^ T cells were extracted from the blood of a healthy donor in accordance with the manufacturer’s instructions (480011, Biolegend, USA). PE anti-human CD3 antibody (BioLegend Cat# 317307, RRID: AB_571912) and KIRAVIA Blue 520™ anti-human CD8 antibody (BioLegend Cat# 344773, RRID: AB_2924543) were used to confirm the identity of the CD8^+^ T cells (Supporting Information Fig. S1A). CD8^+^ T cells were then activated with T-cell activators (11131D, Thermo Fisher, USA) and IL-2 (HY-P7037AF, MCE, China). After 2 days, the activated CD8^+^ T cells formed clusters and exhibited cytotoxicity (Supporting Information Fig. S1B).

Peripheral blood mononuclear cells (PBMCs) were also extracted from the same donor. PBMCs were resuspended in medium containing 1% FBS and incubated in a six-well plate at 37 °C for 1 h. The nonadherent cells were discarded, leaving behind the adherent cells, which were monocytes. These monocytes were differentiated into dendritic cells (DCs) via culture with 50 ng/ml GM-CSF (HY-P78868, MCE, China) and 25 ng/ml IL-4 (HY-P700130AF, MCE, China) for 1 week (Supporting Information Fig. S1C). AGS and HGC27 cells, after heat shock, were cocultured with DCs for 1 day, after which the DCs were induced to become antigen-presenting cells (APCs). AGS and HGC27 cells were plated into 24-well plates, and previously activated CD8^+^ T cells, along with the corresponding APCs, were added for coculture. The viability of the GC cells was monitored every 3 h via a high-content imaging analysis system (Opera Phenix PLUS, PerkinElmer, USA).

### Enzyme-linked immunosorbent assay (ELISA)

ELISA kits for IL-4 (RK00003, ABclonal, China), IL-6 (RK00004, ABclonal, China), IL-10 (RK000012, ABclonal, China), IL-13 (RK00034, ABclonal, China), and TGF-β1 (RK00055, ABclonal, China) were used to measure the optical density (OD) values of these cytokines in serum-free supernatants from an equal number of GC cells in each group, in accordance with the instructions provided by the manufacturer. The OD values were detected at a wavelength of 450 nm, and the cytokine concentrations were determined via the respective standard curves.

### Immunohistochemistry

The 4-μm-thick sections were dewaxed in xylene and rehydrated through a series of graded alcohols. The activity of endogenous peroxidase in the tissues after antigen repair was blocked by incubation with 3% H_2_O_2_ for 20 min. The primary antibodies used for the IHC were against OTUB2 (Thermo Fisher Scientific Cat# PA5-99680, RRID:AB_2818613), YAP1 (Proteintech Cat# 13584-1-AP, RRID:AB_2218915), WWTR1 (Proteintech Cat# 23306-1-AP, RRID:AB_2721185), TGF-β1 (Proteintech Cat# 21898-1-AP, RRID:AB_2811115), CD163 (Proteintech Cat# 16646-1-AP, RRID:AB_2756528), CD206 (Proteintech Cat# 60143-1-Ig, RRID:AB_2144924), KI67 (Proteintech Cat# 27309-1-AP, RRID:AB_2756525), CD274 (Proteintech Cat# 28076-1-AP, RRID:AB_2881052), CD3 (Proteintech Cat# 17617-1-AP, RRID:AB_1939430), CD8a (Proteintech Cat# 66868-1-Ig, RRID:AB_2882205) and SMAD7 (Proteintech Cat# 25840-1-AP, RRID:AB_2848137). The aforementioned antibody dilution ratio and antigen repair-specific conditions were conducted in accordance with the instructions provided by the manufacturer. The protein expression level was evaluated via Image-Pro Plus software, which employs an integrated optical density (IOD) method that incorporates both the staining area and intensity.

### Plasmid transfection

Flag-YAP, Flag-TAZ, Flag-CD274, Myc-OTUB2, Myc-OTUB2-C51S and His-tagged ubiquitin were all constructed by Shanghai GeneChem Co., Ltd. Seed an appropriate number of HEK-293T cells into a six-well plate. To improve transfection efficiency, the culture medium should be replaced with antibiotic-free complete DMEM medium prior to transfection. The ratio of DNA (µg) to Transfection Reagent (µl) was 1:2 and 1:3. Add 250 µl of Opti-MEM™ Reduced Serum Medium to a 1.5 ml EP tube, then add the DNA and transfection reagent separately and incubate for 5 min. Next, mix the Opti-MEM™ Reduced Serum Medium containing the DNA with the Opti-MEM™ Reduced Serum Medium containing the transfection reagent. After incubating for 20 min, add the complex to the cells. 6 h post-transfection, replace the culture medium with complete DMEM medium containing antibiotics. Continue culturing for 24–28 h before proceeding with protein extraction.

### Co-immunoprecipitation (co-IP)

The cells were treated with RIPA buffer (P0013D; Beyotime, China), which contained protease/phosphatase inhibitors and PMSF. The lysates were then subjected to ultrasonication and centrifugation at 14,000 rpm for 15 min at 4 °C. A portion of the supernatant was retained as the INPUT sample. The remaining lysate was incubated overnight at 4 °C with prebalanced agarose beads (sc-2003, Santa Cruz, USA) and the corresponding primary antibody. The next day, the agarose beads were rinsed five times with RIPA buffer. Finally, SDS loading buffer (P0015B, Beyotime, China) was added, and the agarose was boiled to denature the proteins for western blot analysis.

### Western blot

Following cell lysis, ultrasonication, and centrifugation, the supernatant from the lysate was combined with SDS loading buffer (P0015L, Beyotime, China) and denatured by heating. The proteins were then separated via 10% SDS‒PAGE (P0012A, Beyotime, China) and transferred onto polyvinylidene fluoride (PVDF) membranes (IPVH00010, Merck Millipore, USA). To block nonspecific binding, the membranes were treated with 5% skim milk for 1 h. Subsequently, the membranes were incubated overnight at 4 °C with primary antibodies. The next day, the membranes were incubated with the appropriate secondary antibodies at room temperature for 1 h. Protein expression levels were visualized via an enhanced chemiluminescence (ECL) Plus detection system (P10300, NCM Biotech, China). The primary antibodies utilized in this research included the following: OTUB2 (Thermo Fisher Scientific Cat# PA5-99680, RRID: AB_2818613), YAP1 (Proteintech Cat# 13584-1-AP, RRID:AB_2218915), WWTR1 (Proteintech Cat# 23306-1-AP, RRID:AB_2721185), DYKDDDDK (Proteintech Cat# 66008-4-Ig, RRID:AB_2918475), MYC tag (Proteintech Cat# 60003-2-Ig, RRID:AB_2734122), His-Tag (HA) (Proteintech Cat# 66005-1-Ig, RRID:AB_11232599), TGF-β1 (Proteintech Cat# 21898-1-AP, RRID:AB_2811115), GAPDH (Proteintech Cat# 60004-1-Ig, RRID:AB_2107436), SMAD7 (Proteintech Cat# 25840-1-AP, RRID:AB_2848137) and CTGF (Proteintech Cat# 25474-1-AP, RRID:AB_2918089). The protein expression level was evaluated via Image-Pro Plus software, which employs an integrated optical density (IOD) method that incorporates both the staining area and intensity.

### Animal experiments

In total, 24 BALB/c female nude mice (5-week-old) were utilized to create subcutaneous tumor models via AGS and HGC27 cells. GC cells at 70–80% confluence were resuspended in PBS at a density of 4 × 10⁶ cells/100 μl. Then, 100 μl of this cell suspension was administered subcutaneously into the armpit of each mouse, forming a small hillock. 24 C57BL/6 female mice (5-week-old, RRID: IMSR_JAX:000664) with intact immune systems, along with the murine GC cell line MFC, were used to explore the role of OTUB2 in the polarization of TAMs within the TME, as well as the suppression of CD8^+^ T-cell infiltration mediated by the increased expression of CD274 in tumor cells. OTUB2 was first overexpressed or knocked down in MFC cells. Prior to cell injection, the skin of C57BL/6 mice was prepared to facilitate the subsequent observation and measurement of subcutaneous tumors. The MFC cells were injected subcutaneously via the same method as described above. The health of the mice, tumor growth, and body weight were monitored regularly. After 3 weeks, the mice were euthanized, the subcutaneous tumors were excised, and photographic records were taken. The samples were then weighed and fixed in paraformaldehyde for subsequent IHC analysis. All animal experiments were approved by the Committee on the Ethics of Animal Experiments of PUMCH (Approval No. XHDW-2022-108).

### Statistical analysis

To ensure reliability, all the experiments were independently conducted at least three times. The quantitative data are presented as the means ± standard deviations, and differences between groups were analyzed via the independent sample *t* test. Categorical variables are presented as counts and percentages, with group differences assessed by Pearson’s χ² test. Survival analysis was conducted via the Kaplan‒Meier method. Statistical analyses and graphing were performed via SPSS 27.0 (RRID:SCR_002865, Chicago, USA) and GraphPad Prism 8.0 (RRID:SCR_002798, CA, USA). A *P* value < 0.05 was considered statistically significant.

## Results

### OTUB2 was overexpressed in GC and linked to poor prognosis in GC patients

**Fig. 1:**
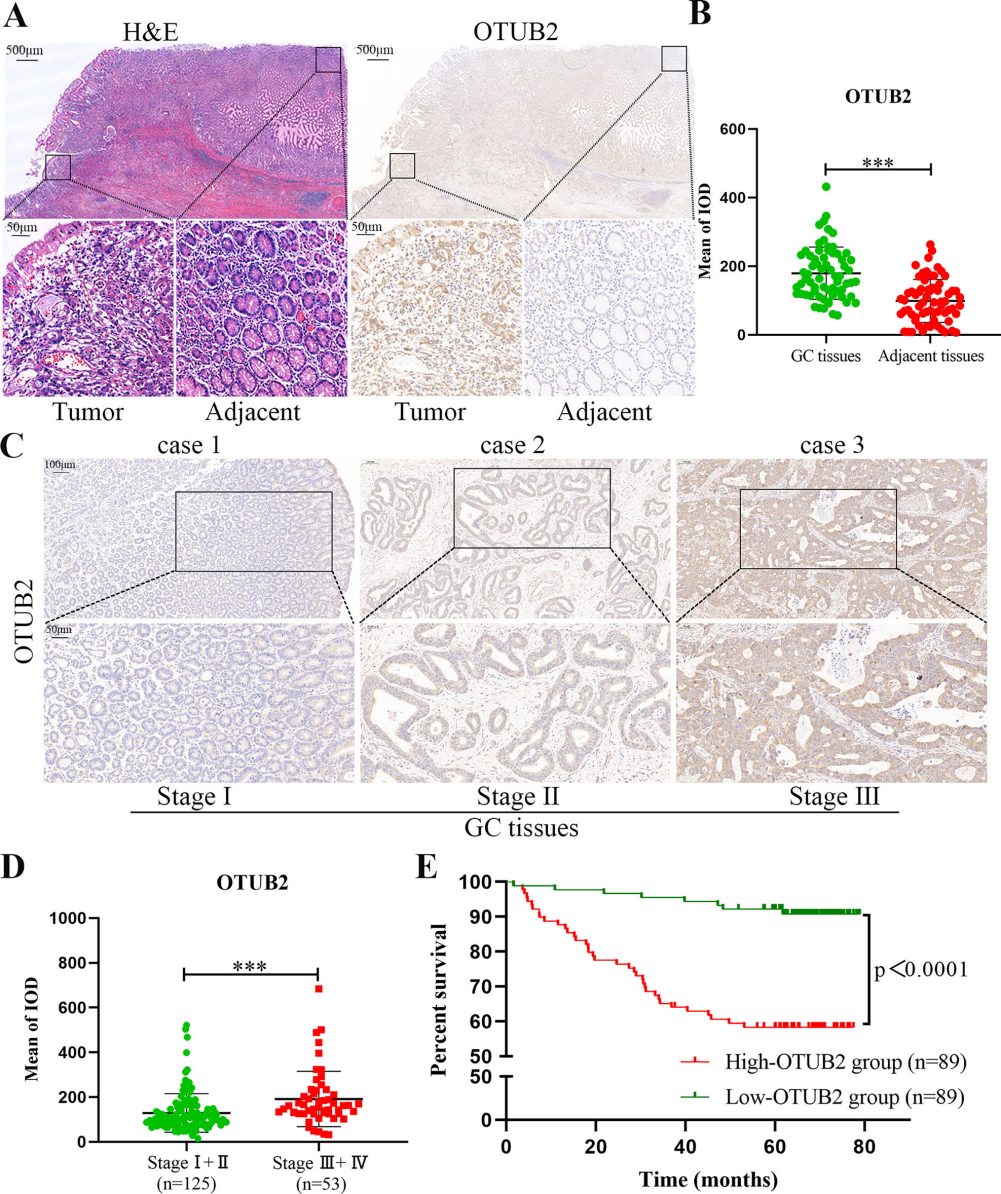
The expression of OTUB2 in GC and its relationship with the prognosis of patients with GC. **A** The expression of OTUB2 in cancer foci and adjacent normal tissues of patients with GC (scale bars: 500 µm and 50 µm). **B** Immunohistochemical (IHC) scores of OTUB2 in 63 paired GC tissues and adjacent normal tissues. **C** The expression of OTUB2 in different clinical stages of GC (scale bars: 100 µm and 50 µm). **D** IHC scores of OTUB2 in different clinical stages of GC (*n* = 178). **E** GC patients were divided into high and low groups according to the IHC score of OTUB2, and the difference in postoperative survival time between the two groups was analyzed (*n* = 178). ***P < 0.001.

The expression of OTUB2 was first verified in GC tissues and their corresponding adjacent tissues. IHC revealed that the level of OTUB2 expression was markedly greater in GC tissues than in adjacent tissues (Fig. 1A). Further analysis of the IHC scores for OTUB2 in paired GC and adjacent tissues revealed significant upregulation of OTUB2 in the GC tissues (Fig. 1B). Moreover, OTUB2 expression not only exceeded that in adjacent tissues but also increased progressively with the clinical stage of GC (Fig. 1C). IHC analysis of postoperative samples from 178 GC patients with complete follow-up data revealed that elevated OTUB2 expression was associated with advanced clinical stage (Fig. 1D) and correlated with shorter long-term survival (Fig. 1E). Additionally, a comprehensive evaluation of 241 GC patients indicated that high OTUB2 expression was often linked to larger tumor size, worse T and N stages, and more advanced clinical stages (Tables 1 and 2). These results indicate that OTUB2 serves as a critical prognostic biomarker in GC, underscoring the need for further investigation into its role in tumor progression and underlying molecular mechanisms.

**Table 1:** Relationship between clinicopathological characteristics and expression of OTUB2 in gastric cancer (*n* = 63).

	Expression of OTUB2		*P* value
	High (*n* = 32)	Low (*n* = 31)	
Gender			
Male	20	22	0.4760
Female	12	9	
Age (years)			
<55	6	7	0.7073
≥55	26	24	
BMI			
<24	15	17	0.5271
≥24	17	14	
Tumor location			
Upper third	7	6	0.6750
Middle third	15	12	
Lower third	10	13	
Tumor size (cm)			
≤3	17	25	0.0205*
>3	15	6	
Tumor T stage			
1 + 2	16	28	*<*0.001***
3 + 4	16	3	
Tumor N stage			
0	13	21	0.0309*
1 + 2 + 3	19	10	
AJCC stage			
I + II	20	28	0.0095**
III + IV	12	3	
Differentiation			
Well-moderately	10	9	0.8475
Poorly	22	22	

Statistical significance was determined by *t* test and chi-square test. *P < 0.05, **P < 0.01, ***P < 0.001.

**Table 2:** Relationship between clinicopathological characteristics and expression of OTUB2 in gastric cancer (*n* = 178).

	Expression of OTUB2		*P* value
	High (*n* = 89)	Low (*n* = 89)	
Gender			
Male	67	60	0.160
Female	22	29	
Age (years)			
<55	19	28	0.087
≥55	70	61	
BMI			
<24	56	47	0.112
≥24	33	42	
Smoking history			
Yes	40	34	0.224
No	49	55	
Tumor location			
Upper third	15	10	0.163
Middle third	30	22	
Lower third	44	57	
Tumor T stage			
1	19	56	*<*0.001***
2	14	17	
3	24	12	
4	32	4	
Tumor N stage			
0	31	62	*<*0.001***
1	14	11	
2	15	8	
3	29	8	
AJCC stage			
I + II	45	80	*<*0.001***
III + IV	44	9	
Differentiation			
Well-moderately	27	28	0.500
Poorly	62	61	

Statistical significance was determined by *t* test and chi-square test. ***P < 0.001.

### Upregulated OTUB2 in GC cells enhanced the polarization of M2 TAMs

**Fig. 2:**
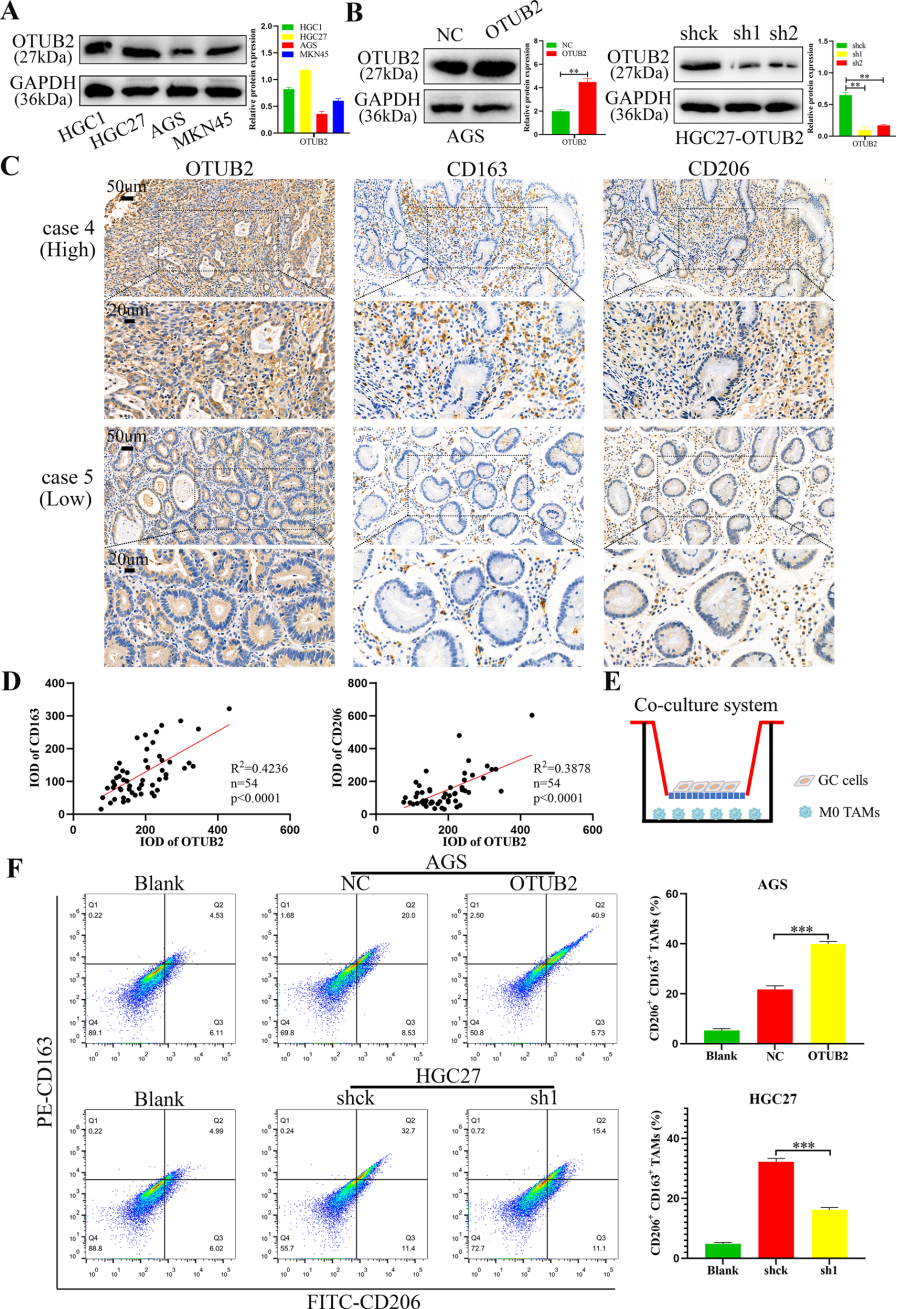
Upregulated OTUB2 in GC cells enhanced the polarization of M2 TAMs. **A** The baseline expression of OTUB2 in four GC cell lines. **B** Western blotting was used to detect the overexpression and knockdown efficiency of OTUB2 in GC cell lines. **C** IHC was used to identify the infiltration of M2 TAMs (CD163 and CD206) in GC tissues with different OTUB2 expression levels (scale bars: 50 µm and 20 µm). **D** The correlations between OTUB2 and CD163 and CD206 expression were analyzed via IHC scores. **E** Schematic diagram of the noncontact coculture of GC cells and M0 macrophages. **F** Flow cytometry was used to detect the proportion of M2 TAMs (CD163 and CD206) after coculture. **P < 0.01, ***P < 0.001.

The baseline expression of OTUB2 in GC cell lines was evaluated via western blot analysis (Fig. 2A). AGS cells were selected for OTUB2 overexpression, whereas HGC27 cells were chosen for OTUB2 knockdown. Immunoblotting confirmed successful overexpression of OTUB2 in AGS cells and significant downregulation of OTUB2 in HGC27 cells (Fig. 2B). Knockdown of OTUB2 notably reduced the proliferation of HGC27 cells, whereas overexpression of OTUB2 markedly enhanced the growth of AGS cells, as shown by CCK-8 and colony formation assays (Supporting Information Fig. S2A, B). Moreover, via flow cytometry, overexpressed OTUB2 facilitated cell cycle transition and decreased the rate of apoptosis in AGS cells, whereas decreased OTUB2 repressed the cell cycle and increased cell apoptosis in HGC27 cells (Supporting Information Fig. S2C, D). These findings suggest that OTUB2 overexpression directly accelerates GC progression by reducing apoptosis and promoting cell cycle progression.

A previous study reported that USP12 triggered the NF-κB pathway by deubiquitinating PPM1B, increasing M2 TAMs infiltration within the TME of lung cancer [21]. In line with these findings, we examined OTUB2 expression and M2 TAMs infiltration (CD163 and CD206) in GC samples. As shown in Fig. 2C, high OTUB2 expression was associated with increased M2 TAMs infiltration in GC tissues. Pearson correlation analysis further demonstrated that OTUB2 expression was strongly positively associated with CD163 and CD206 expression in GC, as determined by the IHC scores (Fig. 2D). To explore the effects of OTUB2 alterations in GC cells on macrophage polarization, we conducted a noncontact coculture experiment. As shown in Fig. 2E, THP-1-derived M0 macrophages were cocultured with GC cells for 48 h, and the classical markers (CD163 and CD206) of M2 TAMs were detected. Flow cytometry revealed that OTUB2 overexpression in AGS cells promoted the polarization of M0 macrophages toward the M2 TAMs phenotype, whereas OTUB2 knockdown in HGC27 cells inhibited this polarization (Fig. 2F). These findings from IHC and coculture experiments demonstrated that OTUB2 upregulation in GCs increased M2 TAMs infiltration in the TME and enhanced M2 TAM polarization in vitro.

### Upregulated OTUB2 inhibited the infiltration of CD8^+^ T cells in the GC TME and increased CD274 expression in GC cells

**Fig. 3:**
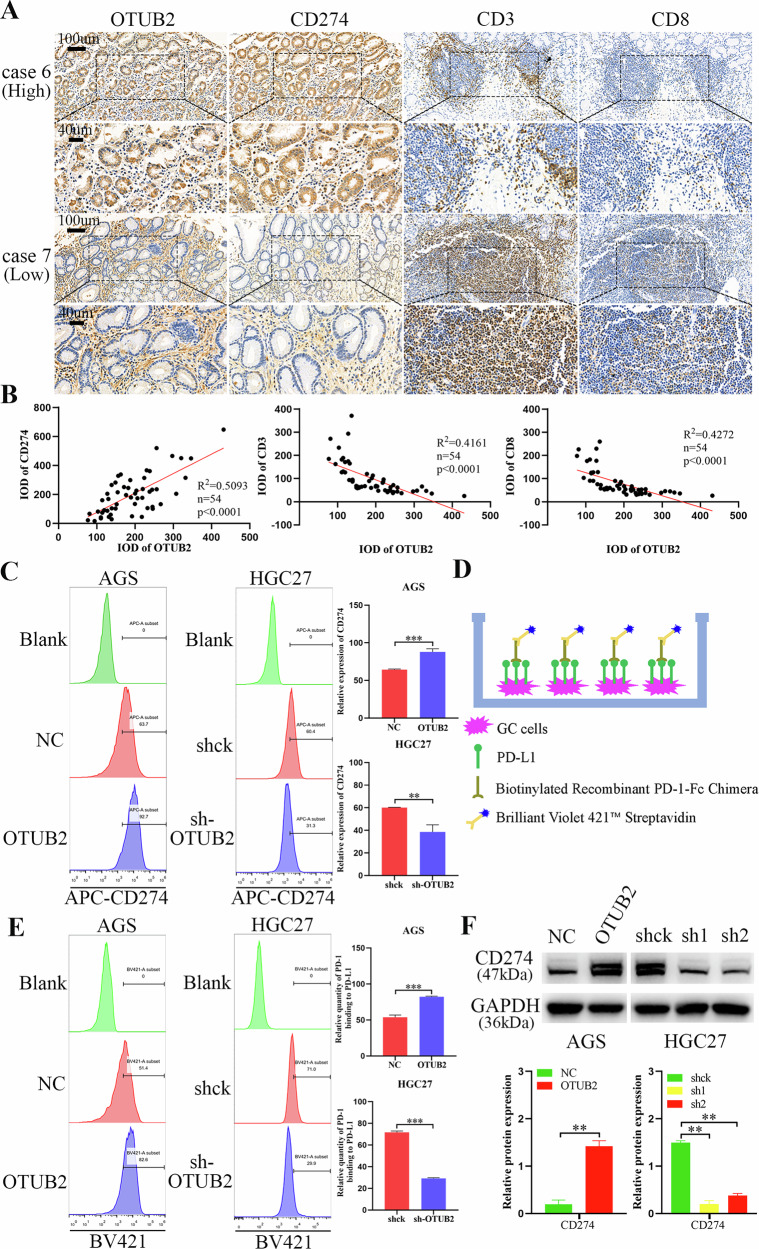
Upregulated OTUB2 inhibited the infiltration of CD8^+^ T cells into the GC TME and increased the expression of CD274 in GC cells. **A** IHC was used to identify the infiltration of CD8^+^ T cells (CD3^+^ and CD8^+^ cells) in GC tissues with different OTUB2 expression levels (scale bars: 100 µm and 40 µm). **B** The correlations between OTUB2 and CD274, CD3 and CD8 expression were analyzed via IHC scores. **C** The expression of CD274 on the surface of GC cells was directly detected via flow cytometry. **D** Schematic diagram of the indirect detection of CD274 expression on the surface of GC cells. **E** CD274 expression on the surface of GC cells was indirectly detected via flow cytometry. **F** Western blotting was utilized to detect the regulation of CD274 expression by alterations in OTUB2 in GC cells. **P < 0.01, ***P < 0.001.

Previous research has shown that USP22 inhibits anticancer immunity by deubiquitinating CD274 [22]. On the basis of these findings, we hypothesized that OTUB2 might similarly aggravate GC progression by deubiquitinating CD274. We first examined the expression of OTUB2 and CD274, as well as the infiltration of CD8^+^ T cells (CD3^+^ and CD8^+^ T cells), in GC samples through IHC staining. As shown in Fig. 3A, CD274 expression was elevated in GC tissues with high OTUB2 expression, and CD8^+^ T-cell infiltration was decreased. Pearson correlation analysis further revealed a positive association between OTUB2 and CD274 expression and a negative association between OTUB2 expression and CD8^+^ T-cell infiltration on the basis of the IHC scores for OTUB2, CD274, CD3 and CD8 in each sample (Fig. 3B). Next, flow cytometry was used to assess CD274 expression in GC cells following OTUB2 overexpression or knockdown. As shown in Fig. 3C, OTUB2 upregulation significantly increased CD274 expression on the surface of AGS cells, whereas OTUB2 downregulation decreased CD274 expression on HGC27 cells. An indirect detection method was also employed to validate the regulatory effects of OTUB2 on CD274 in GC cells. As shown in Fig. 3D, a recombinant biotin-conjugated PD-1 antibody was added to the GC cells which were then streptavidin labeled with Brilliant Violet 421™ (BV421) to indirectly detect CD274 expression via the BV421 intensity. The data revealed that BV421 intensity increased with OTUB2 upregulation, suggesting that OTUB2 promoted CD274 expression on the surface of AGS cells, facilitating PD-1 binding. Conversely, OTUB2 inhibition decreased the BV421 intensity in HGC27 cells (Fig. 3E).

Finally, the western blot results corroborated the flow cytometry findings, showing that OTUB2 overexpression increased CD274 expression in GC cells (Fig. 3F). These findings suggest elevated OTUB2 expression in GC enhances CD274 expression, reduces CD8^+^ T-cell infiltration in the TME, and may contribute to immune evasion in GC cells.

### Upregulated OTUB2 reinforced the immune evasion of GC cells

**Fig. 4:**
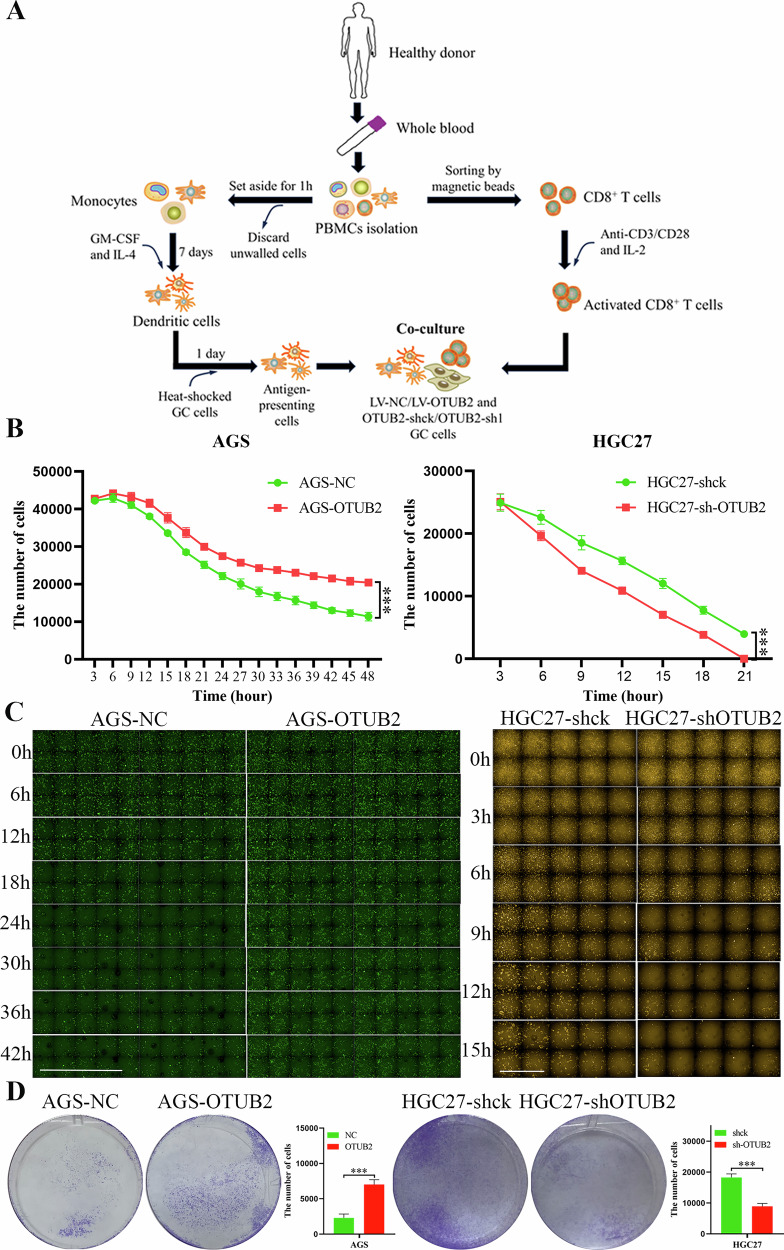
Upregulated OTUB2 reinforced the immune evasion of GC cells. **A** Flow chart of the T-cell killing assay. **B** In GC cells overexpressing OTUB2, immune clearance by CD8^+^ T cells was inhibited. **C** Overexpressed OTUB2 promoted immune evasion and survival in GC cells. **D** Crystal violet staining of GC cells after coculture with CD8^+^ T cells. ns: not significant, ***P < 0.001.

CD8^+^ T cells are the most critical executors of tumor cell clearance in the TME [23, 24]. To confirm the role of OTUB2 in promoting immune evasion in GC cells, a T-cell killing assay was performed. As shown in Fig. 4A, dendritic cell-derived antigen-presenting cells, cytotoxically activated CD8^+^ T cells and GC cells were cocultured. The viability of each group of GC cells was monitored in real time via a high-content imaging analysis system. The results demonstrated that the overexpression of OTUB2 increased the survival of AGS cells in the T-cell killing assay, whereas the knockdown of OTUB2 enhanced the ability of CD8^+^ T cells to kill HGC27 cells (Fig. 4B). Real-time fluorescence imaging further confirmed that OTUB2 overexpression strengthened immune evasion, allowing GC cells to avoid clearance by CD8^+^ T cells (Fig. 4C). Crystal violet staining of GC cells after the T-cell killing assay, as shown in Fig. 4D, revealed that more AGS cells survived after OTUB2 was overexpressed, whereas fewer HGC27 cells survived after OTUB2 was knocked down. These findings indicate that OTUB2 upregulation enhances immune evasion in GC cells, allowing them to evade CD8^+^ T-cell-mediated clearance, thereby exacerbating GC progression.

### Upregulated OTUB2 enhances M2 TAMs polarization by activating the YAP/TAZ pathway

**Fig. 5:**
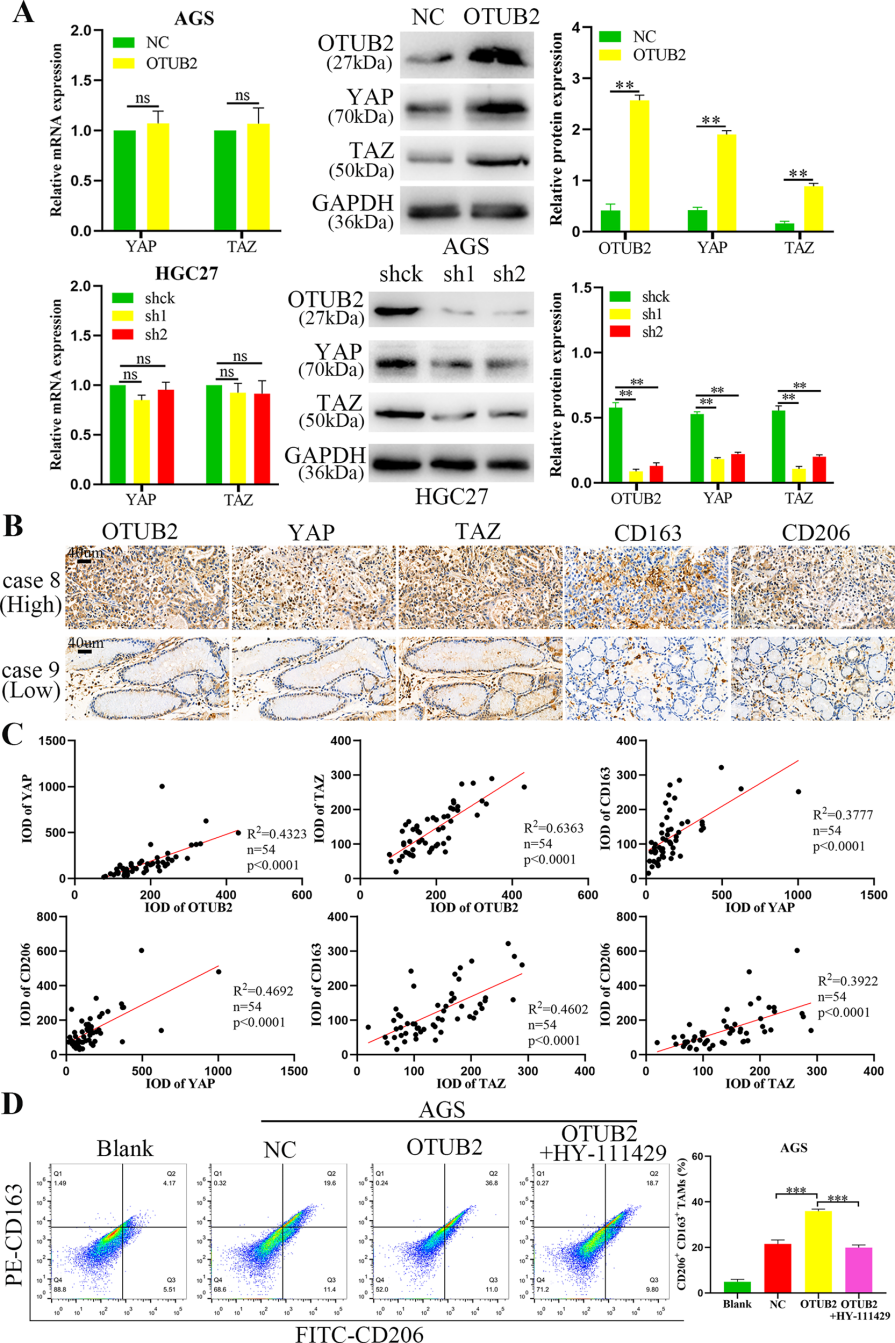
OTUB2 enhanced M2 TAMs polarization through the YAP/TAZ pathway. **A** Overexpressed OTUB2 increased the level of the YAP/TAZ protein and had no effect on its transcription level in AGS cells, whereas downregulated OTUB2 decreased the level of the YAP/TAZ protein and did not affect its transcription level in HGC27 cells. **B** The expression of YAP/TAZ and infiltration of M2 TAMs (CD163 and CD206) in GC tissues with different OTUB2 expression levels were detected via IHC staining (scale bar: 40 µm). **C** The correlations between OTUB2 and YAP/TAZ, YAP/TAZ and CD163 and CD206 expression were analyzed via IHC scores. **D** Inhibition of the YAP/TAZ pathway blocked the promotion of M2 TAMs polarization by GC cells with upregulated OTUB2. ns: not significant, **P < 0.01, ***P < 0.001.

A previous study reported that OTUB2 activated the YAP/TAZ pathway to promote tumor progression [14]. YAP/TAZ, a pair of key downstream effectors of the Hippo pathway, promotes the polarization of M2 TAMs in the TME and accelerates tumor progression [25]. Transcriptome sequencing was performed on HGC27 cells after OTUB2 knockdown (Supporting Information Fig. S3A). KEGG enrichment analysis of differentially expressed genes also revealed that OTUB2 regulate the Hippo signaling pathway (Supporting Information Fig. S3B). In our study, the overexpression of OTUB2 in AGS cells increased the YAP/TAZ protein levels but had no significant effect on their transcription. Conversely, knockdown of OTUB2 in HGC27 cells reduced YAP/TAZ protein levels without substantial changes in mRNA expression, as validated through western blotting and quantitative real-time PCR (Fig. 5A). OTUB2, YAP/TAZ and infiltration of M2 TAMs (CD163 and CD206) were detected in GC samples via IHC. As shown in Fig. 5B, the levels of YAP/TAZ in GC tissues with high expression of OTUB2 were significantly elevated, and the infiltration of M2 TAMs also increased accordingly. Pearson correlation analysis further revealed that OTUB2 was positively correlated with YAP/TAZ expression and M2 TAMs infiltration in GC tissues, as indicated by the IHC scores for OTUB2, YAP/TAZ, CD163 and CD206 (Fig. 5C).

These IHC findings suggest that OTUB2 not only upregulates YAP/TAZ expression but also enhances M2 TAMs infiltration in the GC TME. In a coculture assay of macrophages and GC cells, specific YAP/TAZ inhibitors (HY-111429) were added to OTUB2-overexpressing AGS cells. The results showed that the YAP/TAZ inhibitors blocked the pro-polarization effects of OTUB2 overexpression on M2 TAMs (Fig. 5D). These data confirm that OTUB2 drives M2 TAMs polarization in the TME through activation of the YAP/TAZ pathway in GC cells.

### Upregulated OTUB2 increased YAP/TAZ and CD274 expression by deubiquitinating them

**Fig. 6:**
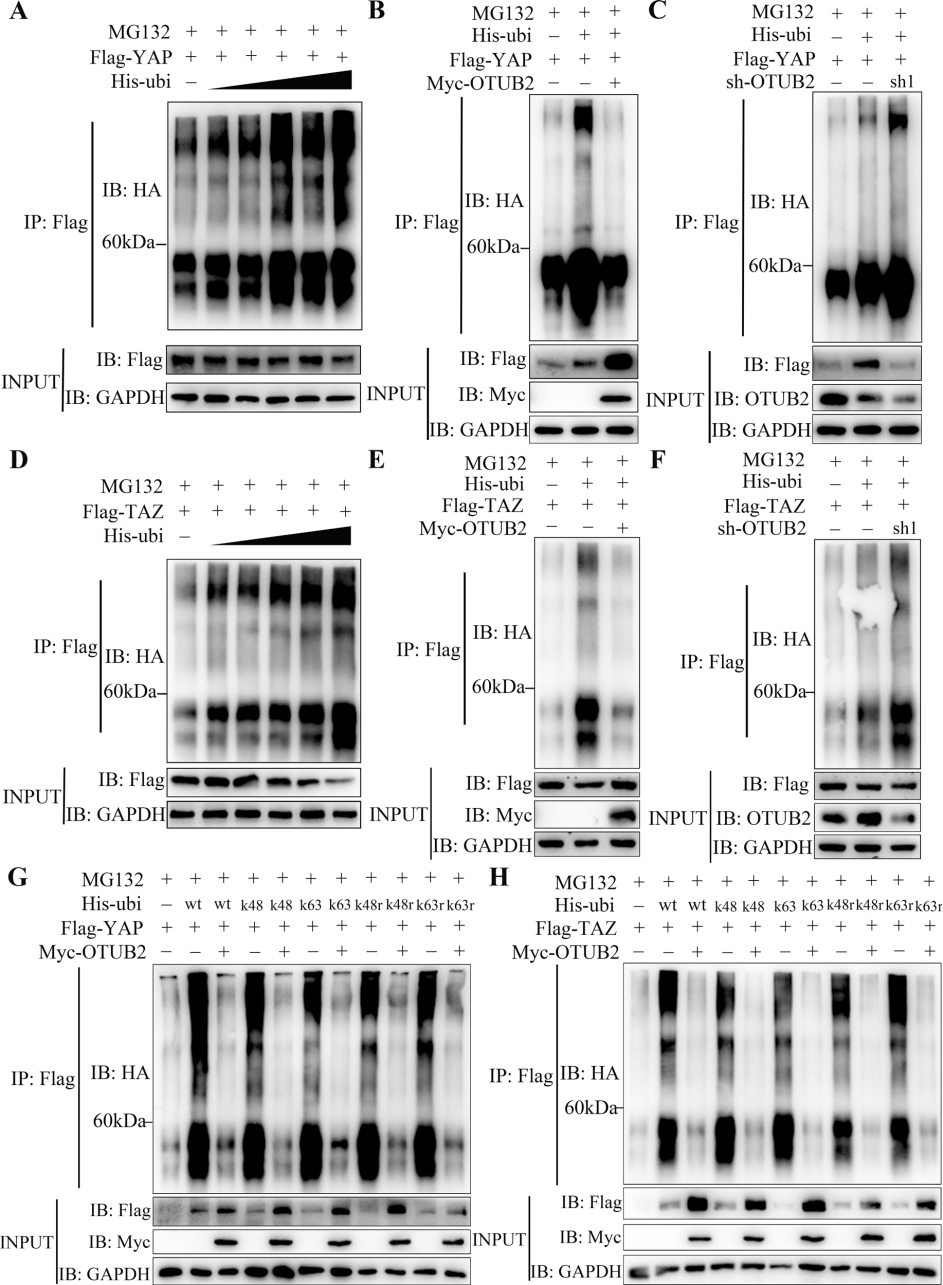
OTUB2 directly deubiquitinates YAP/TAZ via the Lys48 and Lys63 pathways to protect YAP/TAZ from proteasome degradation. **A** The expression of YAP is regulated by the ubiquitin–proteasome system. **B** Overexpressed OTUB2 reduced the ubiquitination of YAP. **C** Downregulation of OTUB2 led to increased ubiquitination of YAP. **D** TAZ expression is mediated by the ubiquitin‒proteasome system. **E** Overexpressing OTUB2 reduced the ubiquitination of TAZ. **F** Downregulation of OTUB2 led to increased ubiquitination of TAZ. **G** OTUB2 deubiquitinated YAP through the Lys48 and Lys63 pathways. **H** OTUB2 deubiquitinated TAZ through the Lys48 and Lys63 pathways.

Co-IP and western blot assays were used to investigate the molecular mechanism through which OTUB2 modulates YAP/TAZ and CD274. Given the role of OTUB2 itself as a DUB, we first confirmed that the expression of YAP/TAZ and CD274 is modulated by the UPS. As shown in Figs. 6A, D and Fig. 8A, the expression of YAP/TAZ and CD274 gradually decreased with increasing ubiquitination. To further explore this, the ubiquitin molecules Myc-OTUB2 and Flag-YAP, Flag-TAZ or Flag-CD274 were cotransfected into HEK-293T cells. The results demonstrated that overexpressed OTUB2 greatly reduced the ubiquitination of YAP/TAZ and CD274 and increased their expression (Figs. 6B, E and 8B), whereas downregulated OTUB2 profoundly enhanced the ubiquitination degradation of YAP/TAZ and CD274, resulting in a decrease in their expression (Figs. 6C, F and Fig. 8C).

**Fig. 7:**
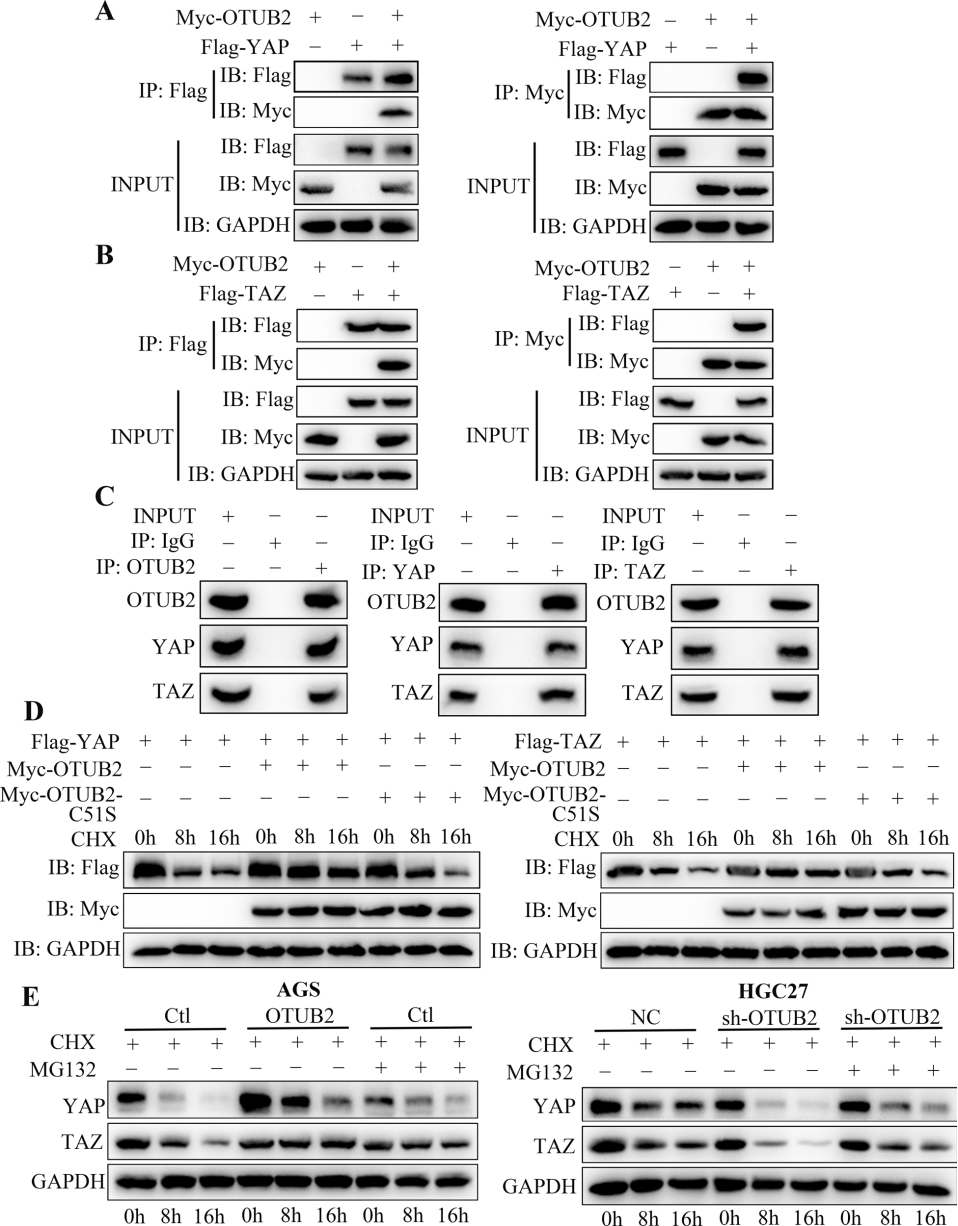
OTUB2 interacted with YAP/TAZ and stabilized its expression. **A** The binding of OTUB2 to YAP was verified by exogenous co-IP. **B** The binding of OTUB2 to TAZ was validated by exogenous co-IP. **C** The binding of OTUB2 to YAP/TAZ was confirmed by endogenous co-IP. **D** OTUB2 prolonged the degradation half-life of the YAP/TAZ protein, whereas the inactive enzyme mutant OTUB2-C51S could not achieve this effect. **E** MG132 rescued the accelerated protein degradation of YAP/TAZ caused by downregulated OTUB2.

**Fig. 8:**
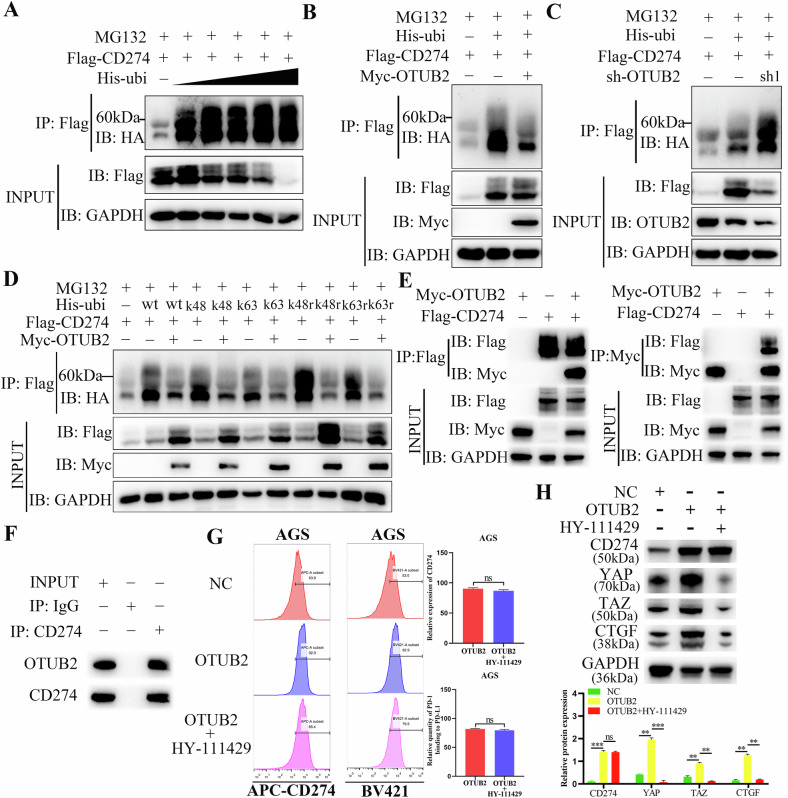
OTUB2 directly deubiquitinates CD274 via the Lys48 and Lys63 pathways to protect CD274 from proteasome degradation. **A** The expression of CD274 is regulated by the ubiquitin‒proteasome system. **B** Overexpressed OTUB2 reduced the ubiquitination of CD274. **C** Downregulation of OTUB2 led to increased ubiquitination of CD274. **D** OTUB2 deubiquitinated CD274 through the Lys48 and Lys63 pathways. **E** The binding of OTUB2 to CD274 was verified by exogenous co-IP. **F** The binding of OTUB2 to CD274 was confirmed by endogenous co-IP. **G** Inhibition of the YAP/TAZ pathway did not affect the expression of CD274 on the surface of GC cells promoted by overexpressed OTUB2. **H** Inhibition of the YAP/TAZ pathway did not affect the expression of CD274 in GC cells promoted by OTUB2 overexpression, as determined via western blotting. ns: not significant, **P < 0.01, ***P < 0.001.

To identify specific deubiquitination sites, ubiquitin molecules with different lysine residues were transfected into HEK-293T cells. The results revealed that OTUB2 mainly deubiquitinated YAP/TAZ and CD274 through lysine residues K48 and K63 (Figs. 6G, H and 8D). Co-IP assays using anti-Flag and anti-Myc antibodies in HEK-293T cells revealed that OTUB2 interacts with YAP/TAZ and CD274, respectively (Figs. 7A, B and 8E). Comparable results were observed in GC cells, confirming that OTUB2 interacts with YAP/TAZ and CD274, preventing their degradation through Lys48- and Lys63-linked ubiquitination (Figs. 7C and 8F).

Furthermore, when cycloheximide (CHX) was added to block protein synthesis in GC cells, OTUB2 overexpression extended the degradation half-life of the YAP/TAZ proteins, whereas the inactive mutant OTUB2-C51S did not exert this effect (Fig. 7D). Adding a proteasome inhibitor (MG132) to GC cells after CHX treatment abolished the YAP/TAZ degradation accelerated by OTUB2 downregulation (Fig. 7E). A previous study reported that YAP/TAZ pathway activation increases CD274 expression [26]. To assess this, YAP/TAZ pathway inhibitors were added to OTUB2-overexpressing AGS cells. Flow cytometry revealed that OTUB2 directly increased CD274 expression on the surface of GC cells (Fig. 8G). The western blot results also confirmed that YAP/TAZ pathway inhibition did not eliminate the OTUB2-mediated increase in CD274 expression (Fig. 8H). These results demonstrate that directly deubiquitinating these proteins enhances their expression and promotes GC progression.

### Upregulated OTUB2 facilitated M2 TAMs polarization by increasing TGF-β1 secretion via inhibiting SMAD7 in GC cells by triggering the YAP/TAZ pathway

**Fig. 9:**
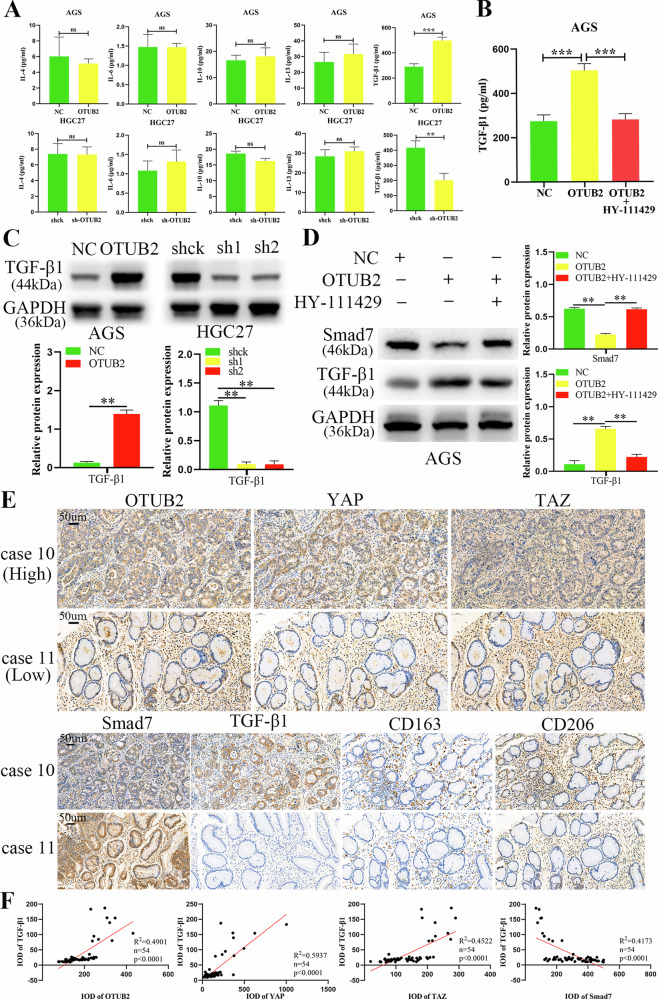
Overexpressed OTUB2 activated the YAP/TAZ pathway to increase TGF-β1 secretion in GC cells. **A** ELISA was used to detect alterations in IL-4, IL-6, IL-10, IL-13 and TGF-β1 in the supernatants of GC cells after OTUB2 was upregulated or downregulated. **B** Inhibition of the YAP/TAZ pathway prevented the promotion of TGF-β1 secretion in GC cells caused by the overexpression of OTUB2. **C** OTUB2 regulated the expression of TGF-β1 in GC cells. **D** Inhibition of the YAP/TAZ pathway prevented the increase in TGF-β1 expression in GC cells caused by the overexpression of OTUB2 by increasing SMAD7. **E** The expression of YAP/TAZ, SMAD7 and TGF-β1 and infiltration of M2 TAMs (CD163 and CD206) in GC tissues with different OTUB2 expression levels were detected via IHC staining (scale bar: 50 µm). **F** The correlations among OTUB2, YAP/TAZ, SMAD7 and TGF-β1 were analyzed via IHC. ns: not significant, **P < 0.01, ***P < 0.001.

To examine the underlying molecular processes through which OTUB2 overexpression in GC cells promotes M2 TAMs polarization, ELISA was used to detect changes in classical cytokines that induce M2 TAMs polarization in the supernatant of GC cells. As shown in Fig. 9A, the levels of IL-4, IL-6, IL-10 and IL-13 in the supernatant were relatively low and did not significantly change after OTUB2 overexpression or knockdown. However, TGF-β1 was abundant in the supernatant and increased significantly with OTUB2 upregulation in AGS cells, whereas its secretion decreased in HGC27 cells following OTUB2 knockdown. These results suggest that elevated TGF-β1 is the key cytokine that promotes M2 TAMs polarization in GC cells overexpressing OTUB2.

To further explore this, the YAP/TAZ pathway inhibitor HY-111429 was added to OTUB2-overexpressing AGS cells. The ELISA results revealed that YAP/TAZ inhibition blocked the OTUB2-induced increase in TGF-β1 secretion (Fig. 9B). Western blot analysis confirmed that OTUB2 upregulation promoted TGF-β1 expression in GC cells (Fig. 9C). The results of chromatin immunoprecipitation revealed that YAP/TAZ does not directly bind to the TGF-β1 promoter region (Supporting Information Fig. S4A, B). Guided by previous literature, which reported that YAP/TAZ can modulate TGF-β1 by regulating SMAD7 [27]. The results of Western blot indicated that OTUB2 promoted TGF-β1 expression via inhibiting SMAD7 by activating the YAP/TAZ pathway (Fig. 9D). Next, the expression of OTUB2, YAP, TAZ, SMAD7, TGF-β1 and M2 TAMs markers (CD163 and CD206) was examined in GC tissue samples. As shown in Fig. 9E, tissues with high OTUB2 expression presented significantly elevated levels of YAP/TAZ and TGF-β1, while decreased SMAD7, alongside increased M2 TAMs infiltration in the TME. Pearson correlation analysis further revealed a significant positive relationship between OTUB2 and YAP/TAZ expression and TGF-β1 levels, while a negative relationship between SMAD7 and TGF-β1 in GC tissues, as determined by IHC scores (Fig. 9F).

### In vivo experiments confirmed that OTUB2 promoted M2 TAMs infiltration by activating the YAP/TAZ pathway to increase TGF-β1 secretion and inhibited CD8^+^ T cell infiltration by increasing CD274 expression in GC cells

**Fig. 10:**
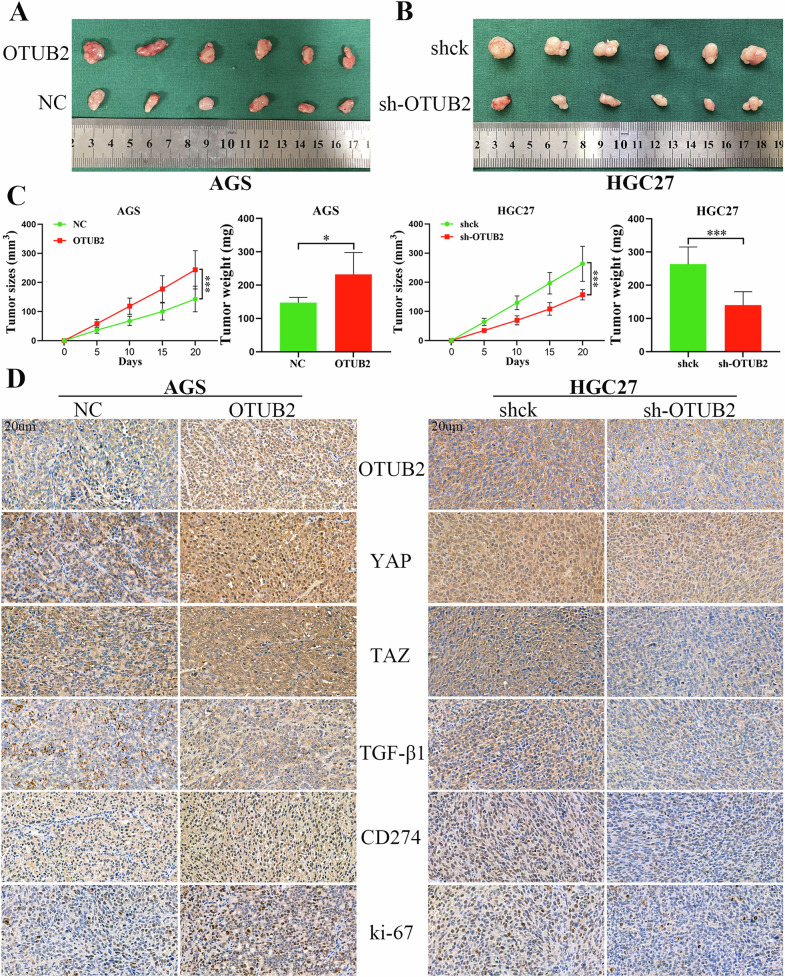
OTUB2 overexpression promoted the growth of GC cells in vivo. **A** The image presents the subcutaneous tumors of AGS cells after overexpressing OTUB2. **B** The image presents the subcutaneous tumors formed from HGC27 cells after OTUB2 was downregulated. **C** The volume changes and weight differences of the subcutaneous tumors. **D** IHC staining of OTUB2, YAP/TAZ, TGF-β1, CD274 and ki-67 in subcutaneous tumors from nude mice (scale bar: 20 µm). *P < 0.05, ***P < 0.001.

The subcutaneous tumor-bearing model was constructed in nude mice via AGS and HGC27 cells in which OTUB2 was overexpressed or knocked down. In vivo experiments revealed that OTUB2 overexpression profoundly promoted AGS cell growth, whereas OTUB2 knockdown inhibited the proliferation of HGC27 cells (Fig. 10A–C). IHC staining revealed that OTUB2 overexpression increased the expression of YAP/TAZ, TGF-β1 and CD274 in GC cells (Fig. 10D).

**Fig. 11:**
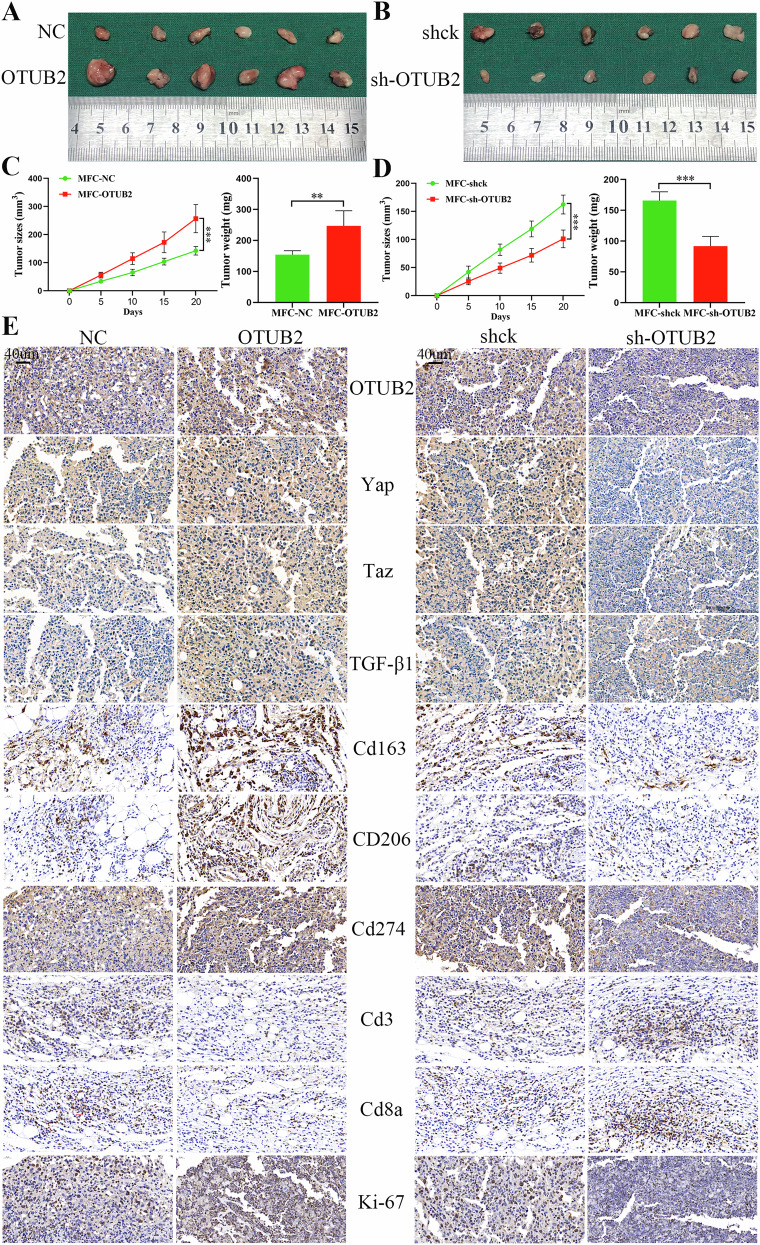
Overexpressed OTUB2 enhanced the infiltration of M2 TAMs and inhibited the infiltration of CD8^+^ T cells in the C57BL/6 mouse model. **A** The image presents the subcutaneous tumors of MFC cells after overexpressing OTUB2. **B** The image presents the subcutaneous tumors formed from MFC cells after OTUB2 was downregulated. **C** Upregulated OTUB2 facilitated the growth of MFC cells. **D** Downregulated OTUB2 suppressed the growth of MFC cells. **E** IHC staining of OTUB2, Yap/Taz, TGF-β1, CD274, and Ki-67 and infiltration of M2 TAMs (Cd163 and CD206) and CD8^+^ T cells (Cd3 and Cd8a) in the subcutaneous tumors of C57BL/6 mice (scale bar: 40 µm). **P < 0.01, ***P < 0.001.

To further verify the effects of alterations in OTUB2 in GC cells on the polarization of M2 TAMs in the TME and the infiltration of CD8^+^ T cells in vivo, a model was established in which C57BL/6 mice with intact immune systems and mouse forestomach carcinoma (MFC) cells were used. MFC cells that successfully upregulated and downregulated OTUB2 were injected subcutaneously into C57BL/6 mice (Supporting Information Fig. S5A, B). The growth pattern of the tumors was consistent with the results in nude mice. Upregulated OTUB2 facilitated the growth of MFC cells (Fig. 11A, C), whereas downregulated OTUB2 repressed the proliferation of MFC cells (Fig. 11B, D). After IHC staining for OTUB2, YAP, TAZ, TGF-β1, and CD274 and the infiltration of M2 TAMs (CD163 and CD206) and CD8^+^ T cells (CD3 and CD8) in the TME, OTUB2 promoted M2 TAMs infiltration by activating the YAP/TAZ pathway to increase TGF-β1 secretion and inhibited the infiltration of CD8^+^ T cells by increasing CD274 expression in MFC cells (Fig. 11E). These in vivo findings indicate that OTUB2 overexpression in GC cells enhances M2 TAMs infiltration in the TME via activation of the YAP/TAZ pathway and increased TGF-β1 secretion. Simultaneously, OTUB2 promotes CD274 expression in GC cells, which inhibits CD8^+^ T-cell infiltration, highlighting OTUB2 as a key target for reprogramming the TME and for potential immunotherapy strategies.

### M2 TAMs enhanced the malignant biological behaviors of GC cells

**Fig. 12:**
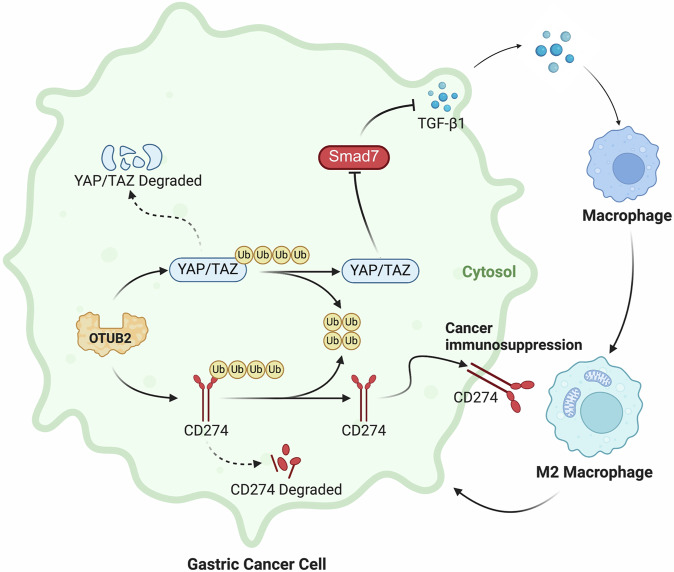
OTUB2 promotes M2 TAMs polarization and CD274 expression in GC progression. Molecular schematic diagram showing that OTUB2 overexpression enhances M2 TAMs polarization and increases the expression of CD274 in GC cells, thereby synergistically aggravating the progression of GC.

The viability of GC cells was assessed after coculture with M2 TAMs stimulated with IL-4 and IL-13 (Supporting Information Fig. S6A). As shown in Supporting Information Fig. S6B, M2 TAMs significantly enhanced GC cell proliferation (Fig. 12). Flow cytometry analysis indicated that M2 TAMs facilitated the transition of GC cells from the G0–G1 phase to the S phase (Supporting Information Fig. S6C). When GC cells were subjected to oxidative stress from H_2_O_2_, M2 TAMs reduced apoptosis and increased the survival ability of GC cells (Supporting Information Fig. S6D). These observations emphasize the significant role of M2 TAMs infiltration in the TME in exacerbating the progression of GC.

## Discussion

China has consistently reported a high incidence of GC, largely attributed to environmental factors, dietary practices, and the prevalence of *Helicobacter pylori* infection [6]. Additionally, the low prevalence of screening and the nonspecific symptoms associated with early-stage GC indicate that approximately 80% of patients are diagnosed at an advanced stage, significantly contributing to the high mortality rates associated with this disease in China [4]. Therefore, exploring the etiology and mechanisms of GC initiation and progression, identifying potential therapeutic targets, and enhancing comprehensive treatment strategies are highly clinically important. Moreover, developing and refining treatment plans that benefit advanced GC patients is both urgent and necessary.

The advent of immune checkpoint inhibitors (ICIs) represents a major breakthrough in the treatment of patients with advanced cancer, becoming the first-line therapy for advanced GC and significantly prolonging patient survival [28, 29]. The current ICIs that target PD-L1 are mainly the following four drugs: durvalumab [30], avelumab [31], atezolizumab [32] and sugemalimab [33]. A phase Ib/II clinical trial assessing the safety and efficacy of durvalumab, either as monotherapy or in combination with tremelimumab, in patients with chemotherapy-refractory advanced gastric and esophagogastric junction adenocarcinoma reported a relatively low objective response rate (ORR) in this population, regardless of treatment modality [9]. Conversely, another phase II clinical trial assessing the safety and tolerability of ceralasertib combined with durvalumab demonstrated enhancements in ORR, progression-free survival, and overall survival in the combination group [34]. The findings from these clinical trials indicate that there is still a lack of effective and defined molecular subtypes of GC to guide treatment regimen selection, resulting in limited benefits for patients. Therefore, identifying novel molecular subtypes for immunotherapy and investigating the mechanisms of immune evasion in GC are crucial. Such efforts will not only facilitate precise immunotherapy regimen selection but also enhance the efficacy of ICIs in treating GC. Accumulating evidence has demonstrated that the TME, which comprises all nonmalignant stromal cells that encapsulate tumor cells, significantly influences the genetic and epigenetic changes in tumor cells and plays a vital regulatory role in tumor initiation, development, and metastasis [35, 36]. TAMs, as key effector cells of nonspecific immunity, are pivotal in shaping the immune microenvironment of tumors. Research has demonstrated that the interaction between tumor cells and TAMs profoundly impacts tumor progression, including in GC. TAMs have greatly advanced immunotherapy for GC and remain a key focus in clinical translational research [37, 38].

Ubiquitination and deubiquitination are comprehensive, dynamic, and highly specific biological processes, and any imbalance in these processes can profoundly impact key cellular mechanisms, including protein degradation, DNA repair, transcriptional activation, and gene silencing [11]. OTUB2, a member of the OTU family of deubiquitinating enzymes, has been extensively linked to the progression of malignant tumors. Ren et al. identified OTUB2 as a promising target for improving the efficacy of ICIs by inhibiting the ubiquitination and degradation of PD-L1, leading to elevated PD-L1 expression and enabling tumor cells to evade immune clearance [39]. Furthermore, OTUB2 has been implicated in enhancing aerobic glycolysis in colorectal cancer (CRC) cells, promoting malignant progression by inhibiting the ubiquitination of PKM2, making it a promising candidate target for the metabolic reprogramming of CRC [40]. Our previous study also investigated the role of OTUB2 in GC, demonstrating that it activates the AKT pathway by preventing the ubiquitination and degradation of KRT80, thus accelerating GC progression [16]. However, its effects on reprogramming the TME and regulating immune evasion in GC remain unclear. Additionally, the role of OTUB2 in promoting immune escape and mediating interactions between GC cells and TAMs requires further exploration.

In this study, IHC staining of paired clinical samples first revealed that OTUB2 expression in GC tissues was significantly higher than that in adjacent noncancer tissues. Moreover, OTUB2 expression increased with increasing clinical stage in GC samples. Patients with elevated OTUB2 expression presented larger tumor diameters, advanced TNM stages, higher clinical stages, and shorter overall survival times than patients with normal OTUB2 expression, according to clinicopathological and follow-up data. Previous studies have revealed that USP12 activates the NF-κB pathway by deubiquitinating PPM1B, increasing the percentage of M2 TAMs in the TME of lung cancer [21]. Similarly, we investigated the association between OTUB2 expression and M2 TAMs infiltration (CD163 and CD206) in GC samples. Our results revealed that GC tissues with high OTUB2 expression had increased M2 TAMs infiltration, with a significant positive correlation between these factors. Flow cytometry further demonstrated that OTUB2 overexpression in GC cells significantly elevated the proportion of M2 TAMs in coculture experiments. Previous research has also suggested that OTUB2 activates the YAP/TAZ pathway, thereby promoting tumor progression [14]. As crucial downstream mediators of the Hippo pathway, YAP/TAZ have been implicated in enhancing M2 TAMs polarization in the TME and accelerating tumor growth [25]. Our Western blot and qRT‒PCR analyses revealed that OTUB2 overexpression in GC cells substantially increased YAP/TAZ protein levels without affecting their transcription. IHC analysis of GC tissues confirmed that YAP/TAZ protein levels were markedly elevated in OTUB2-high tissues, which also exhibited increased M2 TAMs infiltration. Furthermore, treatment of AGS cells with YAP/TAZ-specific inhibitors revealed that blocking the YAP/TAZ pathway inhibited the OTUB2-induced polarization of M2 TAMs. While M1 markers of TAMs were not tested in this research, future studies should examine the broader impact of OTUB2 on the M1/M2 spectrum to fully map the immune landscape.

Mechanistically, OTUB2 was found to increase YAP/TAZ protein levels by reducing their ubiquitination, particularly through modifications at the K48 and K63 lysine residues. However, given the complexity of the ubiquitin code, we cannot exclude the possibility that OTUB2 may also modulate non-canonical ubiquitin linkages (e.g., K11, K27, or linear chains). Whether these atypical chains contribute to the fine-tuning of YAP/TAZ or PD-L1 function in gastric cancer warrants further investigation using linkage-specific antibodies or mass spectrometry. Co-IP experiments, both exogenous and endogenous, verified the direct binding of OTUB2 to YAP/TAZ. To further elucidate the molecular mechanisms underlying OTUB2-mediated M2 TAMs polarization in GC cells, we used ELISA to measure changes in classical cytokines secreted by GC cells. TGF-β1 emerged as the key cytokine driving M2 TAMs polarization, as induced by OTUB2-overexpressing GC cells. Importantly, YAP/TAZ-specific inhibitors suppressed the OTUB2-mediated upregulation of TGF-β1 expression and secretion in GC cells. IHC analysis further demonstrated that tissues with high OTUB2 expression had significantly elevated YAP/TAZ and TGF-β1 protein levels, as well as increased M2 TAMs infiltration. Moreover, positive correlations were observed between OTUB2, YAP/TAZ and TGF-β1 expression levels. In vivo experiments confirmed that OTUB2 overexpression activated the YAP/TAZ pathway, increasing TGF-β1 expression in MFC cells and promoting M2 TAMs infiltration in the TME. In turn, coculture assays revealed that M2 TAMs promoted GC cell proliferation by promoting cell cycle progression and suppressing apoptosis. IHC results demonstrated that CD274 expression was increased in GC tissues with elevated OTUB2 expression, whereas the infiltration of CD8^+^ T cells (CD3 and CD8) was reduced in the GC TME. Given the positive correlation between OTUB2 and CD274 expression in GC samples, we further investigated CD274 expression in GC cells with upregulated or downregulated OTUB2. Flow cytometry revealed that OTUB2 overexpression markedly increased CD274 surface expression on GC cells, whereas OTUB2 inhibition reduced CD274 levels. Western blot analysis corroborated these findings, showing that upregulated OTUB2 significantly increased CD274 expression in GC cells.

Since CD274 (PD-L1) on tumor cells primarily interacts with CD279 (PD-1) on T cells, inhibiting T-cell proliferation and cytotoxicity, we conducted T-cell killing assays to confirm the role of OTUB2 in facilitating immune evasion in GC. The results indicated that increased OTUB2 expression enhanced GC cell survival, whereas reduced OTUB2 levels facilitated immune clearance by CD8^+^ T cells. Mechanistically, OTUB2 increased CD274 expression by reducing its ubiquitination, specifically via lysine residues K48 and K63, as confirmed by exogenous and endogenous co-IP experiments that verified the binding of OTUB2 to CD274. Previous studies have proposed that YAP/TAZ pathway activation enhances CD274 expression [26]. However, the flow cytometry and western blot results in our study confirmed that YAP/TAZ pathway inhibition did not affect OTUB2-induced CD274 upregulation in GC cells. In vivo experiments further confirmed that OTUB2 overexpression increased CD274 levels in MFC cells and reduced the infiltration of CD8^+^ T cells (CD3^+^ and CD8^+^ T cells) in the TME. These findings confirmed that OTUB2 directly deubiquitinates CD274, increasing its expression and promoting immune evasion, thereby aggravating GC progression. Despite providing novel insights into the role of OTUB2 in inducing M2 TAM polarization and promoting immune evasion, our study has several limitations. First, the association between OTUB2 expression and immunotherapy efficacy in advanced GC patients was not explored. Second, the potential regulatory role of OTUB2 in modulating the therapeutic effects of ICIs in GC was not investigated in vivo. Further research is needed to explore the clinical importance of OTUB2, especially its involvement in immune evasion and M2 TAMs polarization, to assess its viability as a potential therapeutic target and its impact on immunotherapy outcomes in GC patients.

In addition to immune evasion, the tumor microenvironment plays a pivotal role in supporting tumor angiogenesis, a critical hallmark of cancer progression. Although our current study primarily focused on the regulation of immune responses, the OTUB2-mediated signaling axes we identified likely contribute to gastric cancer angiogenesis. Accumulating evidence indicates that YAP/TAZ signaling acts as a potent driver of angiogenesis [41], directly regulating the transcription of angiogenic factors such as CTGF, CYR61, and VEGF [42]. Furthermore, M2-polarized TAMs, which we found to be significantly increased by OTUB2-mediated TGF-β1 secretion, are well-documented sources of VEGF and matrix metalloproteinases that facilitate vascular remodeling [43]. Therefore, it is reasonable to speculate that OTUB2 overexpression not only orchestrates an immunosuppressive microenvironment but also fosters a pro-angiogenic niche by stabilizing YAP/TAZ and recruiting M2 TAMs [44]. This multifaceted regulation underscores the potential of OTUB2 as a dual-target therapeutic candidate to simultaneously disrupt immune evasion and tumor vascularization.

## Conclusion

In summary, OTUB2 activates the YAP/TAZ pathway by deubiquitinating YAP/TAZ, leading to increased secretion of TGF-β1 in GC cells, which in turn enhances M2 TAMs polarization within the TME. Additionally, OTUB2 elevates CD274 (PD-L1) expression in GC cells by deubiquitinating CD274, thereby inhibiting the immune clearance function of CD8^+^ T cells and promoting immune evasion by GC cells. This study provides important insights and contributes to the understanding of TME reprogramming and the potential for improving immunotherapy in patients with GC.

## Supplementary information

Raw data of western blot

Supplementary information

Supplementary figure 1

Supplementary figure 2

Supplementary figure 3

Supplementary figure 3

Supplementary figure 5

Supplementary figure 6

Supplementary table 1

## Data Availability

The datasets used and/or analyzed during the current study are available from the corresponding author upon reasonable request.
