# Supplementary material for: OTUB2 induces M2 tumor-associated macrophage polarization and increases CD274 expression in gastric cancer cells to aggravate the progression of gastric cancer
Source: Cell Death Dis. 2026 Apr 15;17(1):509. doi: 10.1038/s41419-026-08743-9 (PMC13201613; doi:10.1038/s41419-026-08743-9)
Supplement: Supplementary file 1 — Raw data of western blot [file 41419_2026_8743_MOESM1_ESM.pdf]

Raw data of western blot

Figure 2A：

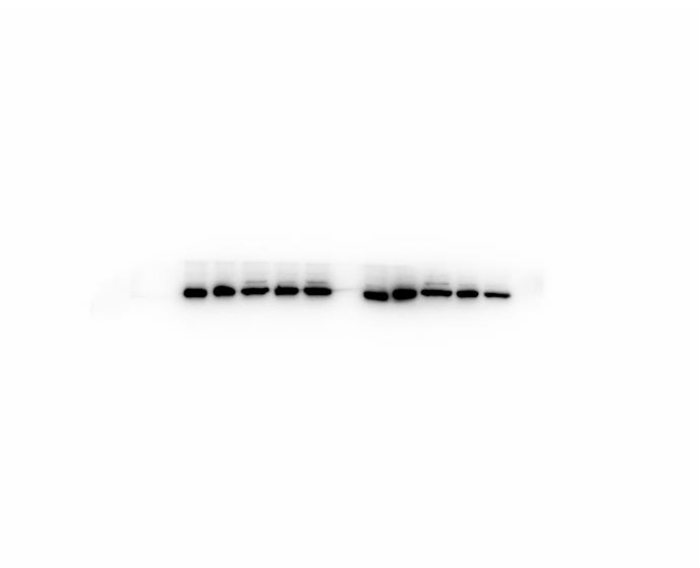

GAPDH

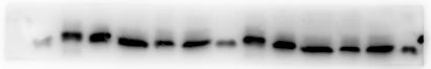

OTUB2

Figure 2B：

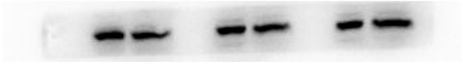

AGS-GAPDH

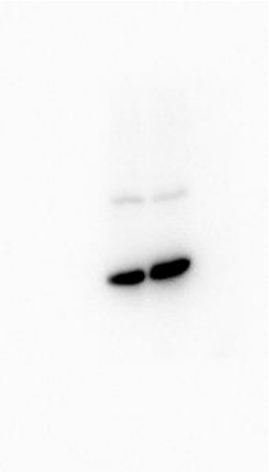

AGS-OTUB2

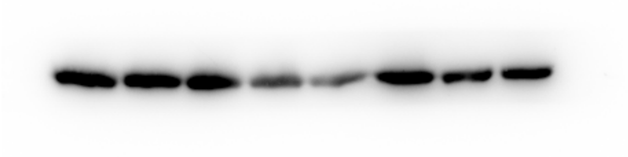

HGC27-GAPDH

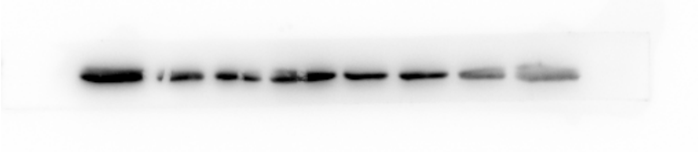

HGC27-OTUB2

Figure 3F：

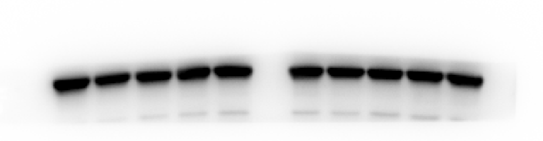

GAPDH

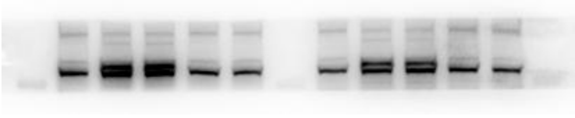

CD274

Figure 5A：

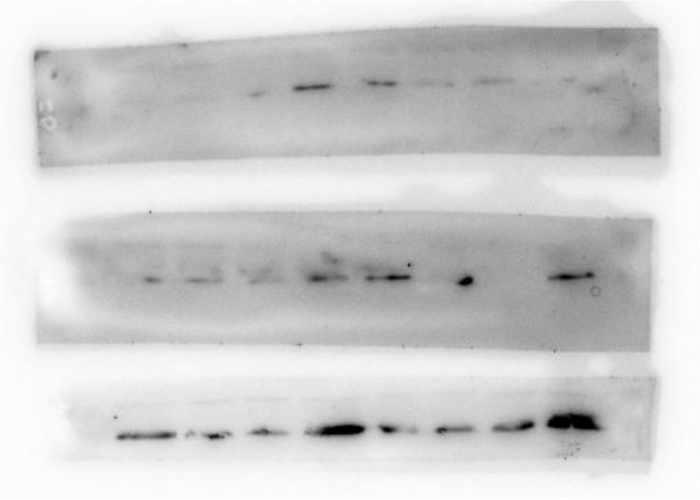

AGS-OTUB2

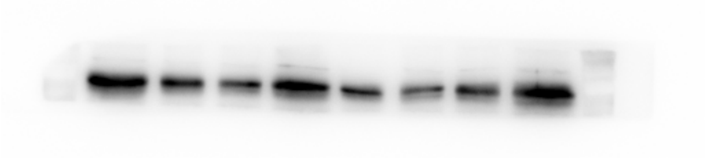

AGS-YAP

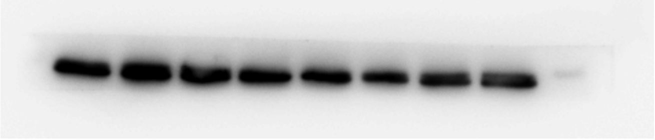

GAPDH

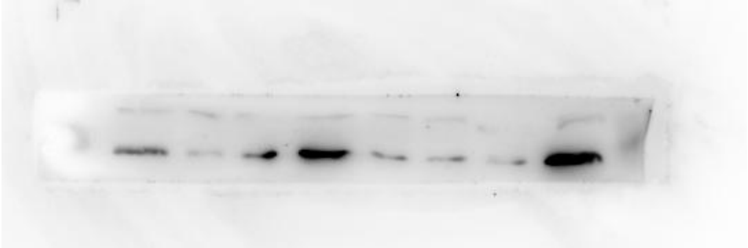

HGC27-OTUB2

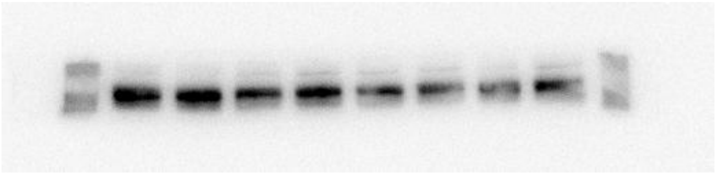

HGC27-YAP

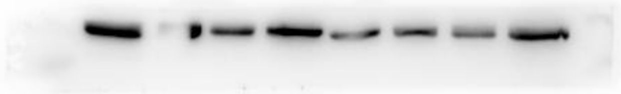

TAZ

Figure 6A：

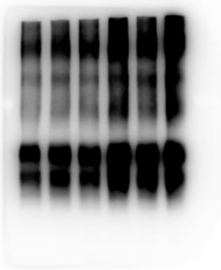

IP-HA

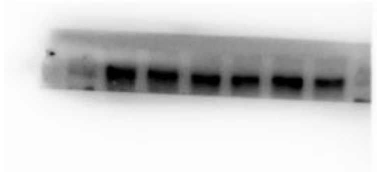

INPUT-FLAG-YAP

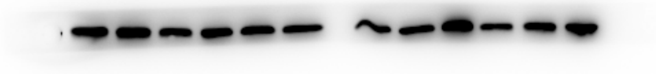

INPUT-GAPDH

Figure 6B：

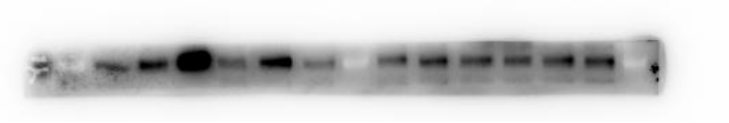

INPUT-FLAG-YAP

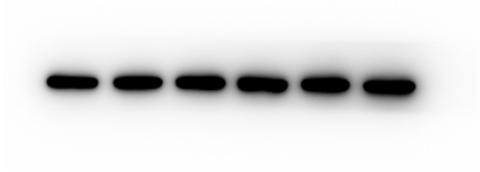

INPUT-GAPDH

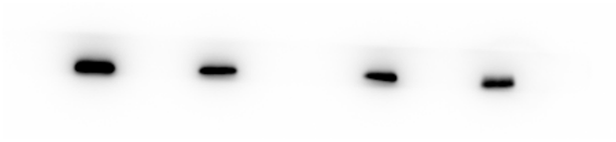

INPUT-myc-OTUB2

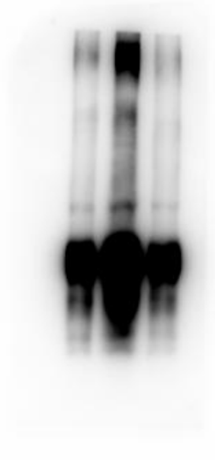

IP-HA

Figure 6C：

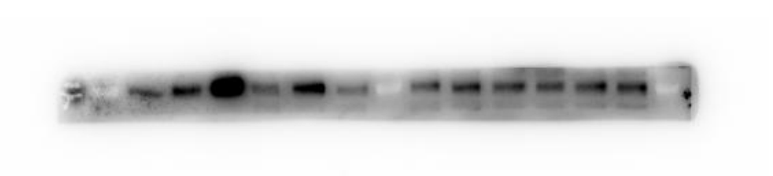

INPUT-FLAG-YAP

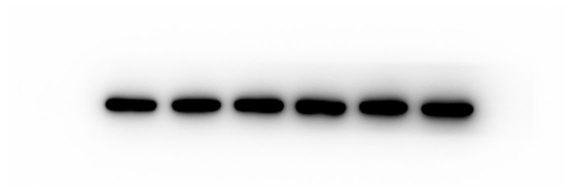

INPUT-GAPDH

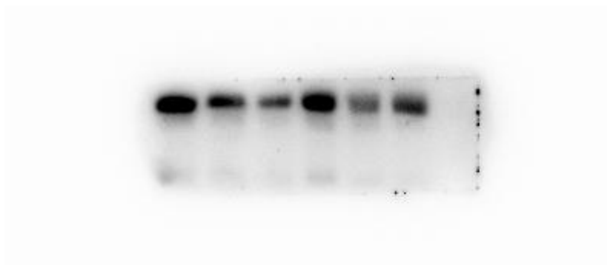

INPUT-OTUB2

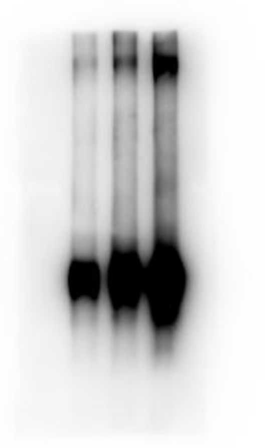

IP-HA-right

Figure 6D:

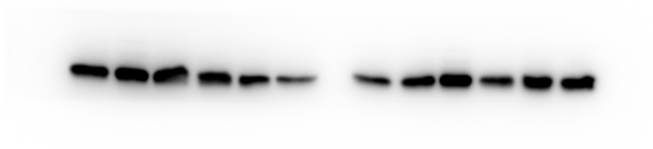

INPUT-FLAG-TAZ

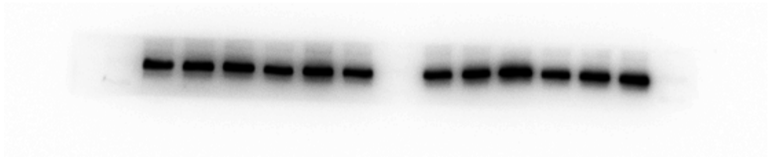

INPUT-GAPDH

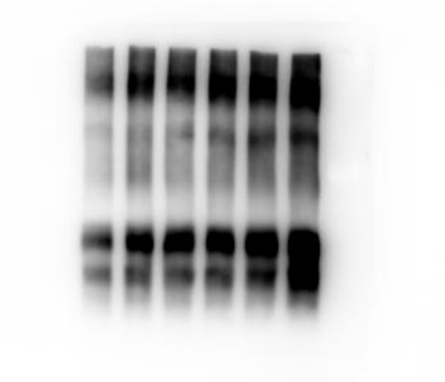

IP-HA

Figure 6E：

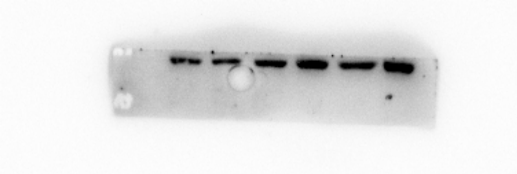

INPUT-FLAG-TAZ

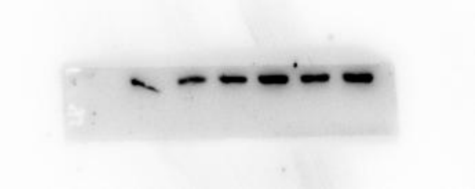

INPUT-GAPDH

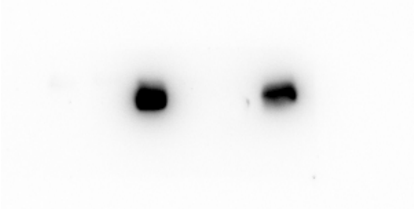

INPUT-myc

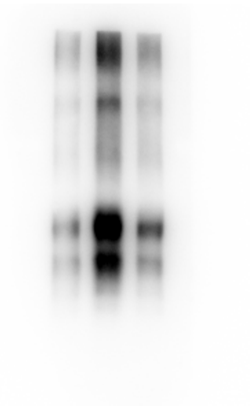

IP-HA

Figure 6F：

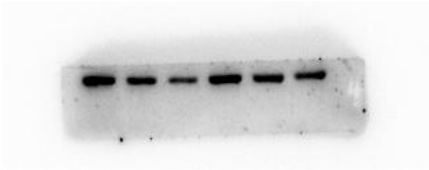

INPUT-FLAG-TAZ

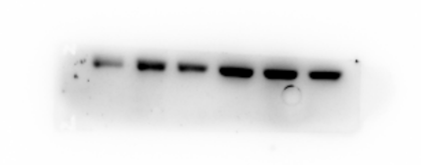

INPUT-GAPDH

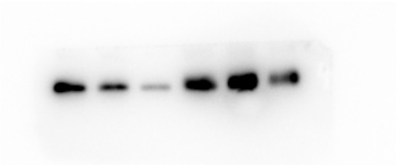

INPUT-OTUB2

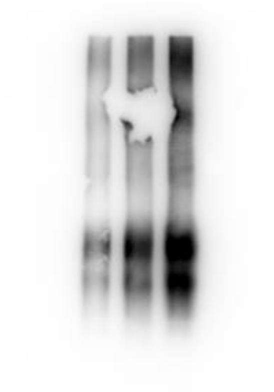

IP-HA

Figure 6G：

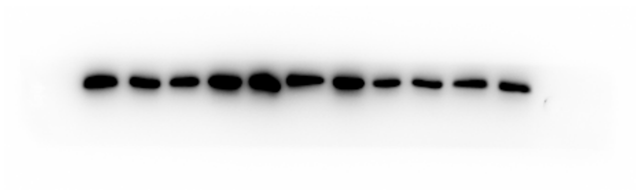

INPUT-GAPDH

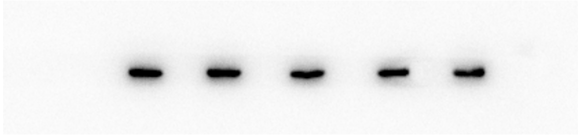

INPUT-myc

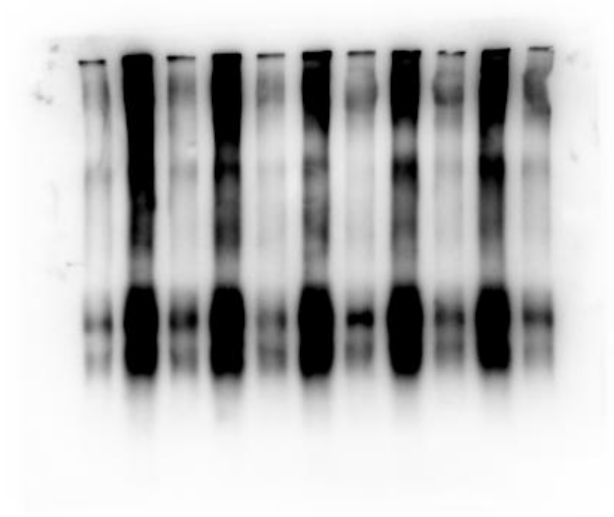

IP-HA

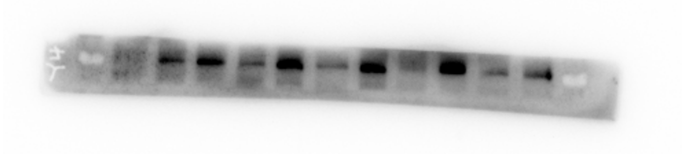

IUPUT-FLAG-YAP

Figure 6H：

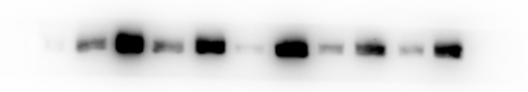

INPUT-FLAG-TAZ

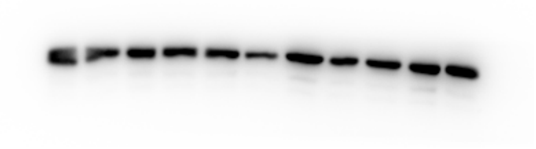

INPUT-GAPDH

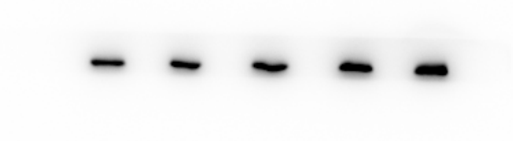

INPUT-myc

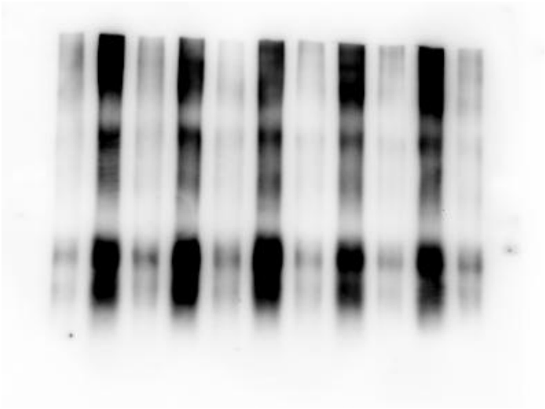

IP-HA

Figure 7A：

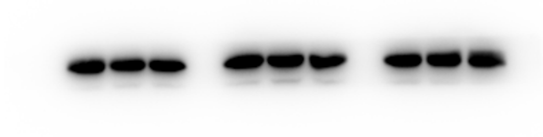

INPUT-GAPDH

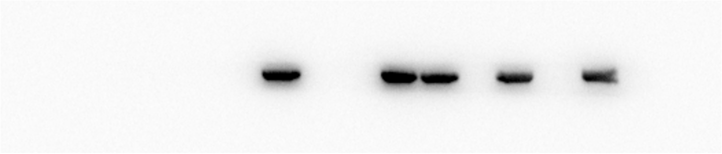

INPUT-MYC

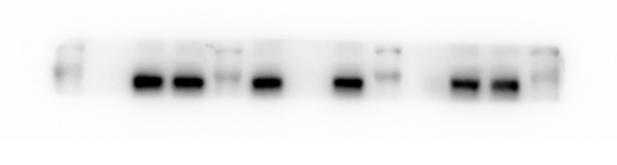

INPUT-YAP

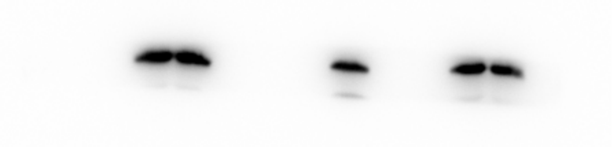

IP-MYC

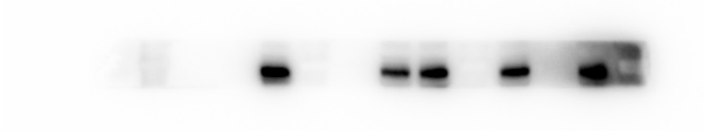

IP-YAP

Figure 7B：

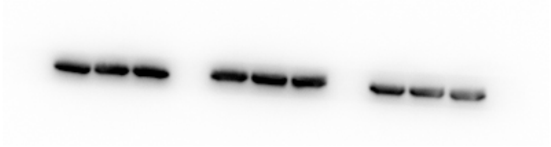

INPUT-GAPDH

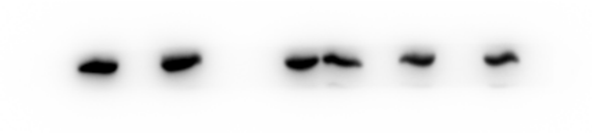

INPUT-MYC

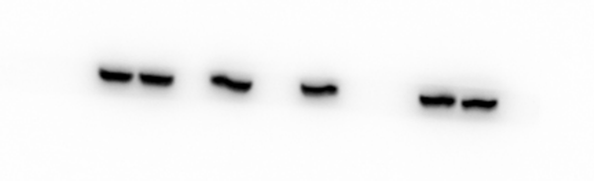

INPUT-TAZ

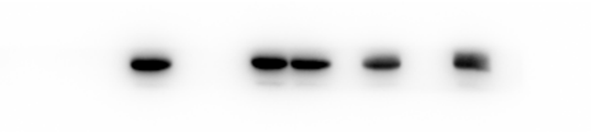

IP-myc

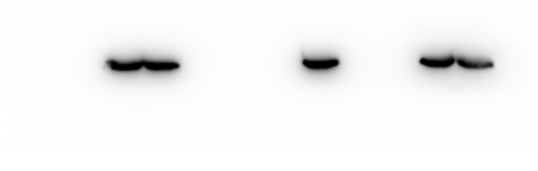

IP-TAZ

Figure 7C：

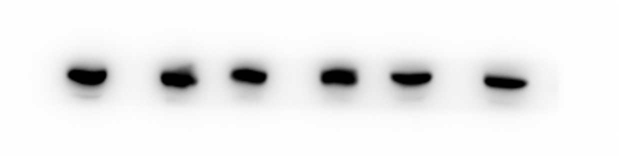

OTUB2

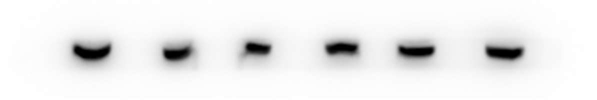

TAZ

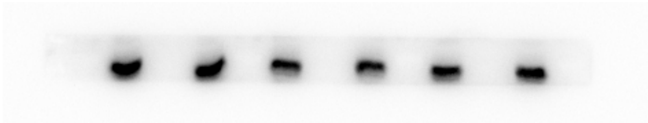

YAP

Figure 7D：

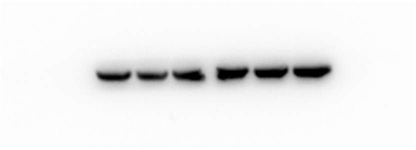

MYC-right

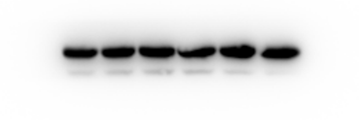

myc-left

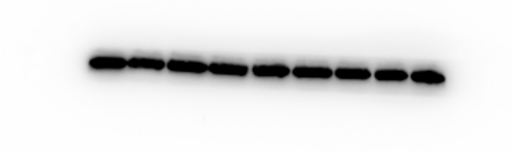

GAPDH-right

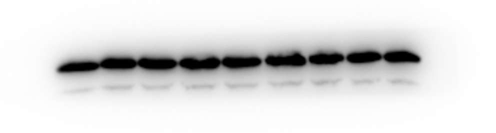

GAPDH-left

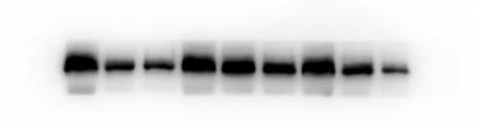

FLAG-YAP

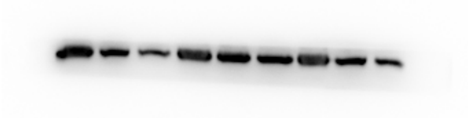

FLAG-taz

Figure 7E：

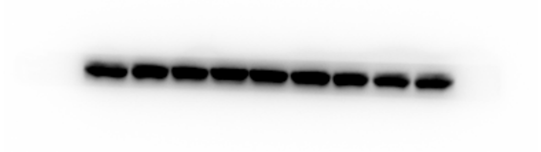

AGS-GAPDH

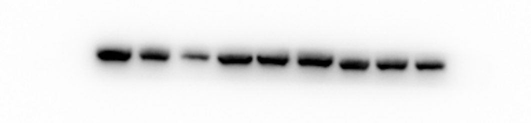

AGS-TAZ

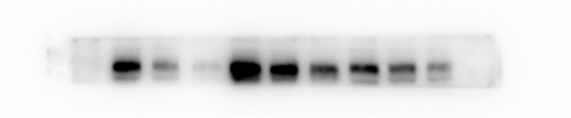

AGS-YAP

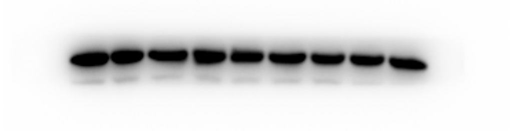

HGC27-GAPDH

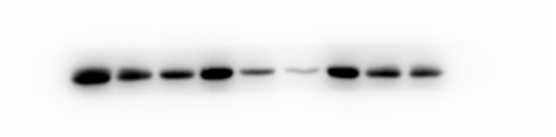

HGC27-TAZ

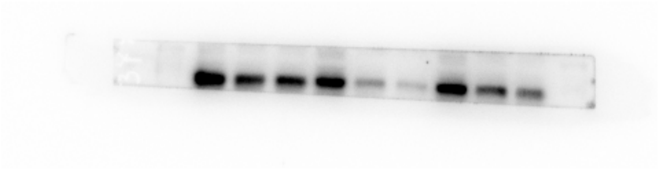

HGC27-YAP

Figure 8A：

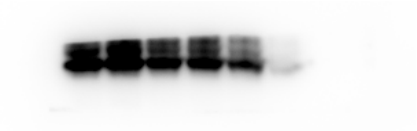

INPUT-FLAG-CD274

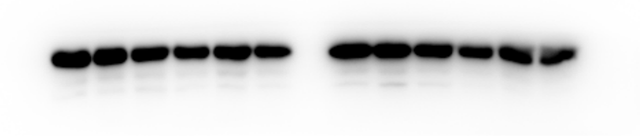

INPUT-GAPDH

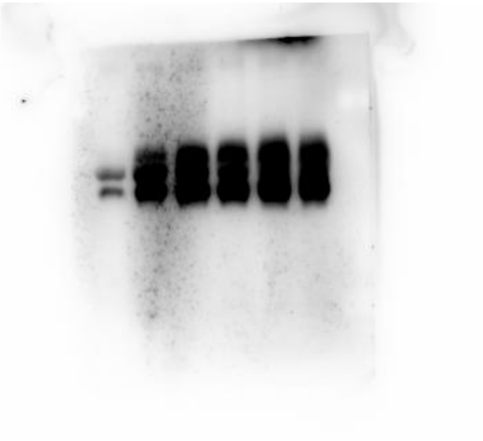

IP-FLAG-CD274

Figure 8B：

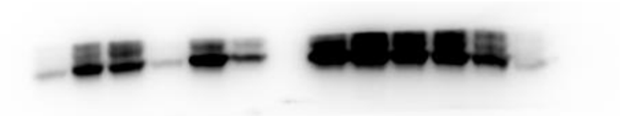

INPUT-FLAG-CD274

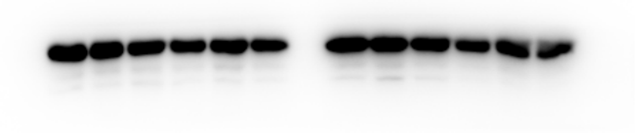

INPUT-GAPDH

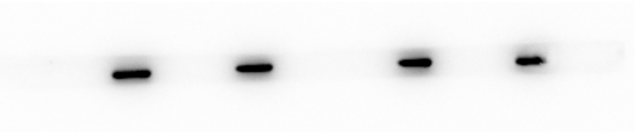

INPUT-myc-OTUB2

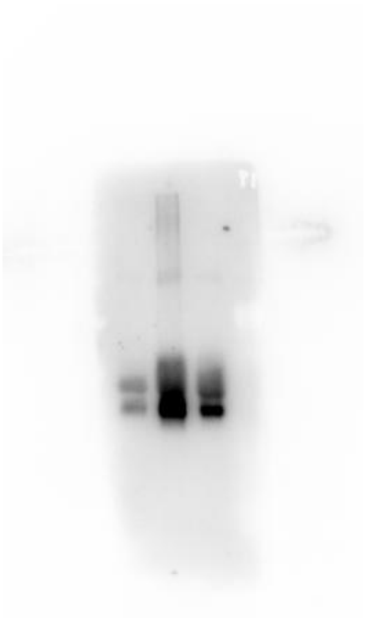

IP-FLAG-CD274

Figure 8C：

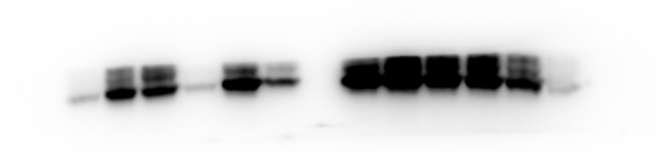

INPUT-FLAG-CD274

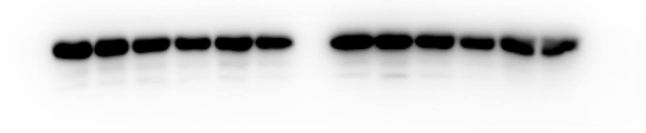

INPUT-GAPDH

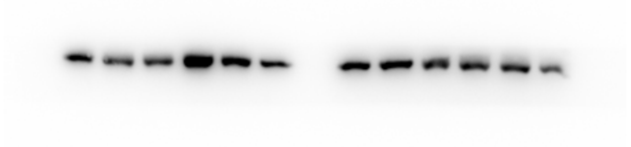

INPUT-OTUB2

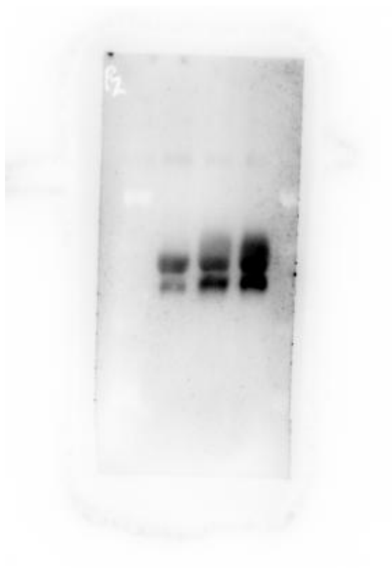

IP-FLAG-CD274

Figure 8D：

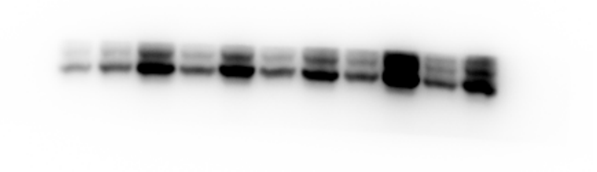

INPUT-FLAG-CD274

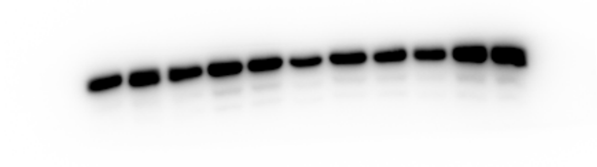

INPUT-GAPDH

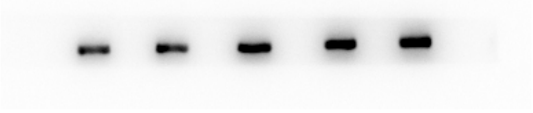

INPUT-MYC-OTUB2

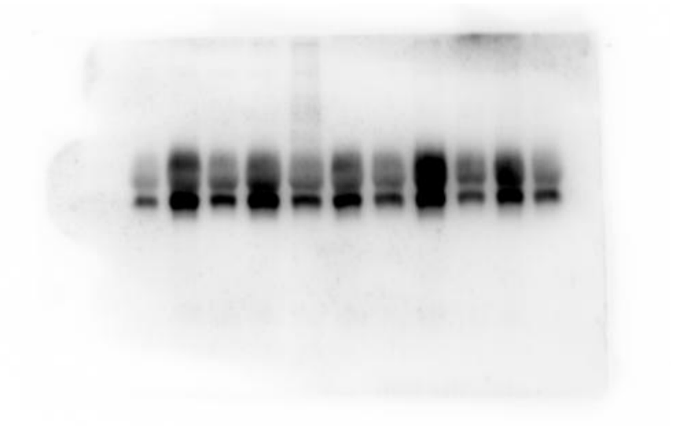

IP-FLAG-CD274

Figure 8E：

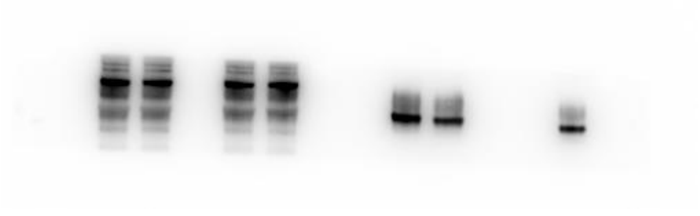

INPUT-FLAG-CD274

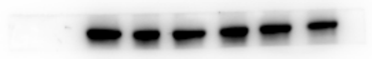

INPUT-GAPDH

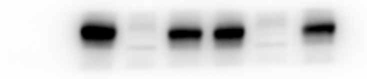

INPUT-MYC-OTUB2

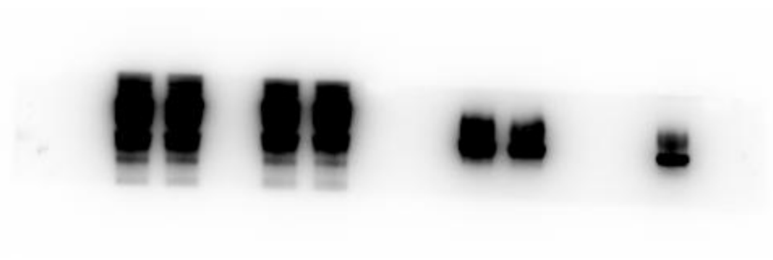

IP-FLAG-CD274

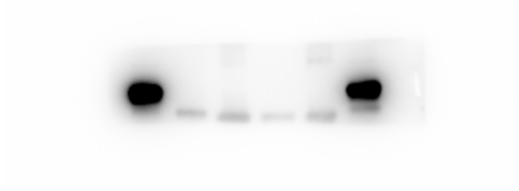

IP-myc-left

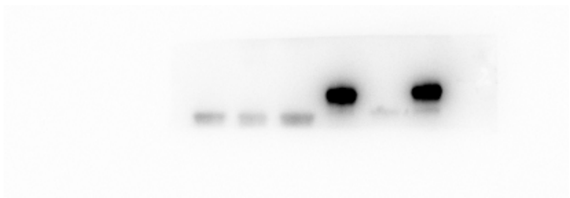

IP-myc-right

Figure 8F:

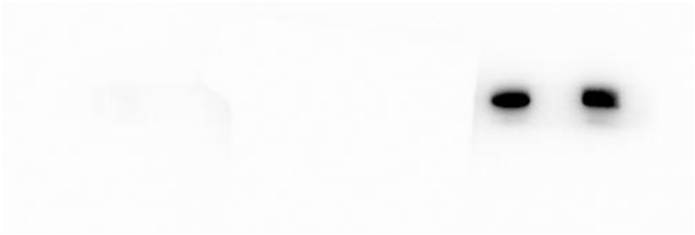

OTUB2

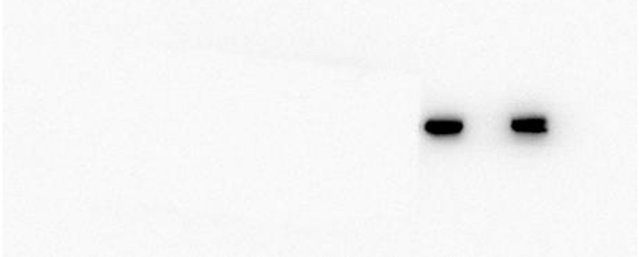

CD274

Figure 8H：

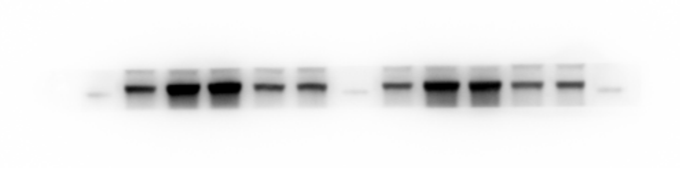

CD274

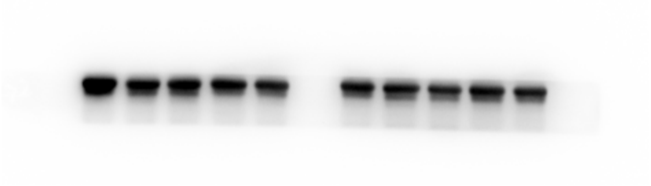

GAPDH

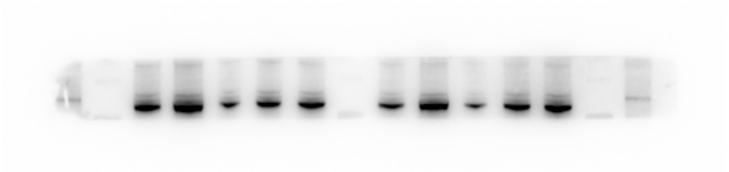

TAZ

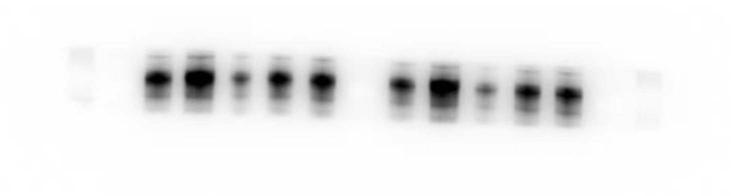

YAP

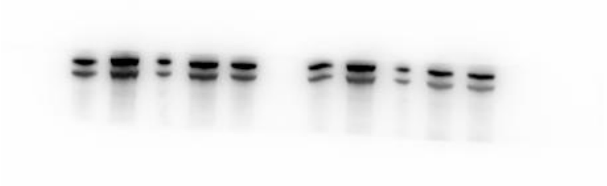

CTGF

Figure 9C:

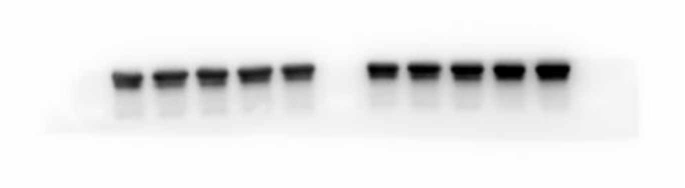

GADPH

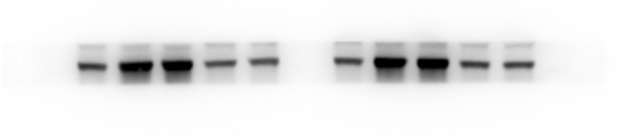

TGF-β1

Figure 9D:

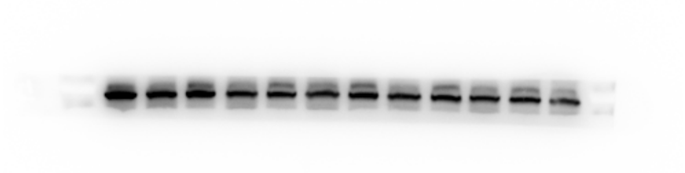

GAPDH

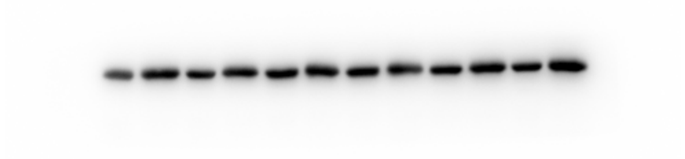

TGF-β1

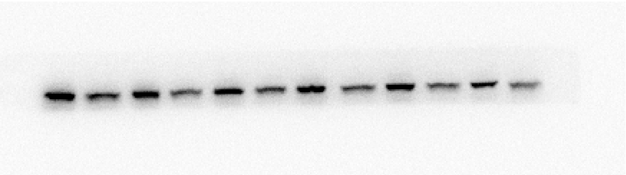

Smad7

Supplementary Figure 3B：

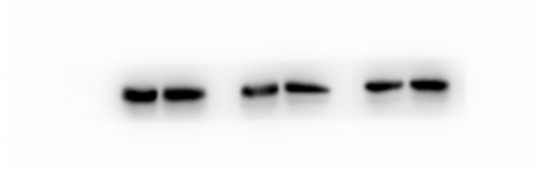

GAPDH-left

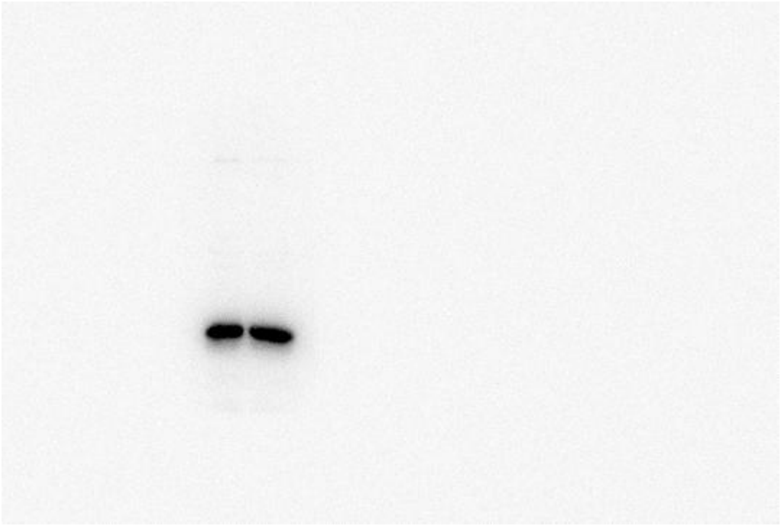

GAPDH-right

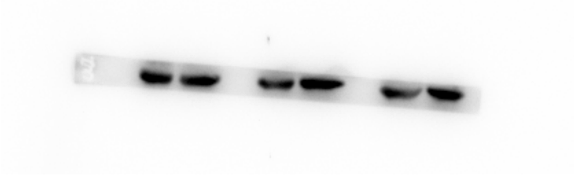

OTUB2-left

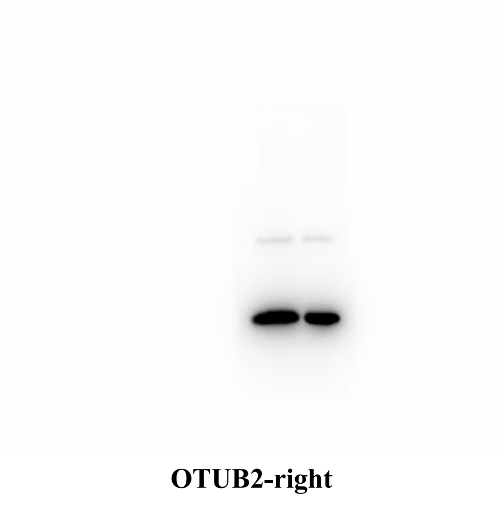

OTUB2-right
