# Supplementary material for: OTUB2 induces M2 tumor-associated macrophage polarization and increases CD274 expression in gastric cancer cells to aggravate the progression of gastric cancer
Source: Cell Death Dis. 2026 Apr 15;17(1):509. doi: 10.1038/s41419-026-08743-9 (PMC13201613; doi:10.1038/s41419-026-08743-9)
Supplement: Supplementary file 2 — Supplementary information [file 41419_2026_8743_MOESM2_ESM.docx]

**Supplementary information**

**Supplementary Figure 1** Morphological alterations of dendritic cells and identification of CD8^+^ T cells. **(A)** The results of CD8^+^ T cells identification by flow cytometry. **(B)** CD8^+^ T cells were activated after adding human T-Activator CD3/CD28 and IL-2 for 2 days. **(C)** Monocytes were induced into dendritic cells after adding GM-CSF and IL-4 for 7 days.

**Supplementary Figure 2** Upregulated OTUB2 promoted the malignant biological behaviors of GC cells. **(A)** CCK-8 assay detected the effects of OTUB2 on the proliferation of GC cells. **(B)** The effects of OTUB2 on GC cells' proliferation and colony formation were detected by colony formation assay. **(C)** The effects of OTUB2 on the cell cycle of GC cells were detected by flow cytometry. **(D)** The effects of OTUB2 on the apoptosis of GC cells were detected by flow cytometry. **p* <0.05, ***p* <0.01, ****p* <0.001

**Supplementary Figure 3** Transcriptome sequencing results of GC cells after knockdown of OTUB2. (**A**) The volcano map of differentially expressed genes between OTUB2 knockdown group and the control group. (**B**) The KEGG enrichment analysis of differentially expressed genes between OTUB2 knockdown group and control group.

**Supplementary Figure 4** The results of chromatin immunoprecipitation in GC cells. (**A**) The qRT-PCR results of TGF-β1 in GC cells using YAP antibody. (**B**) The qRT-PCR results of TGF-β1 in GC cells using TAZ antibody.

**Supplementary Figure 5** Upregulated and downregulated OTUB2 in MFC cells. **(A)** The immunofluorescence image presented the transfection rate of OTUB2 virus in MFC cells (scale bar: 50um). **(B)** Western blot was used to verify the efficiency of upregulated and downregulated OTUB2.

**Supplementary Figure 6** M2 TAMs boosted the malignant biological behaviors of GC cells. **(A)** The schematic diagram of the non-contact co-culture between M2 TAMs and GC cells. **(B)** M2 TAMs promoted the cell proliferation of GC. **(C)** M2 TAMs accelerated the cell cycle transition of GC. **(D)** M2 TAMs decreased the cell apoptosis of GC. ***p* <0.01, ****p* <0.001

**Supplementary Table 1**. The shRNA sequences of OTUB2.
