# Supplementary figures and images for: OTUB2 induces M2 tumor-associated macrophage polarization and increases CD274 expression in gastric cancer cells to aggravate the progression of gastric cancer
Source: Cell Death Dis. 2026 Apr 15;17(1):509. doi: 10.1038/s41419-026-08743-9 (PMC13201613; doi:10.1038/s41419-026-08743-9)

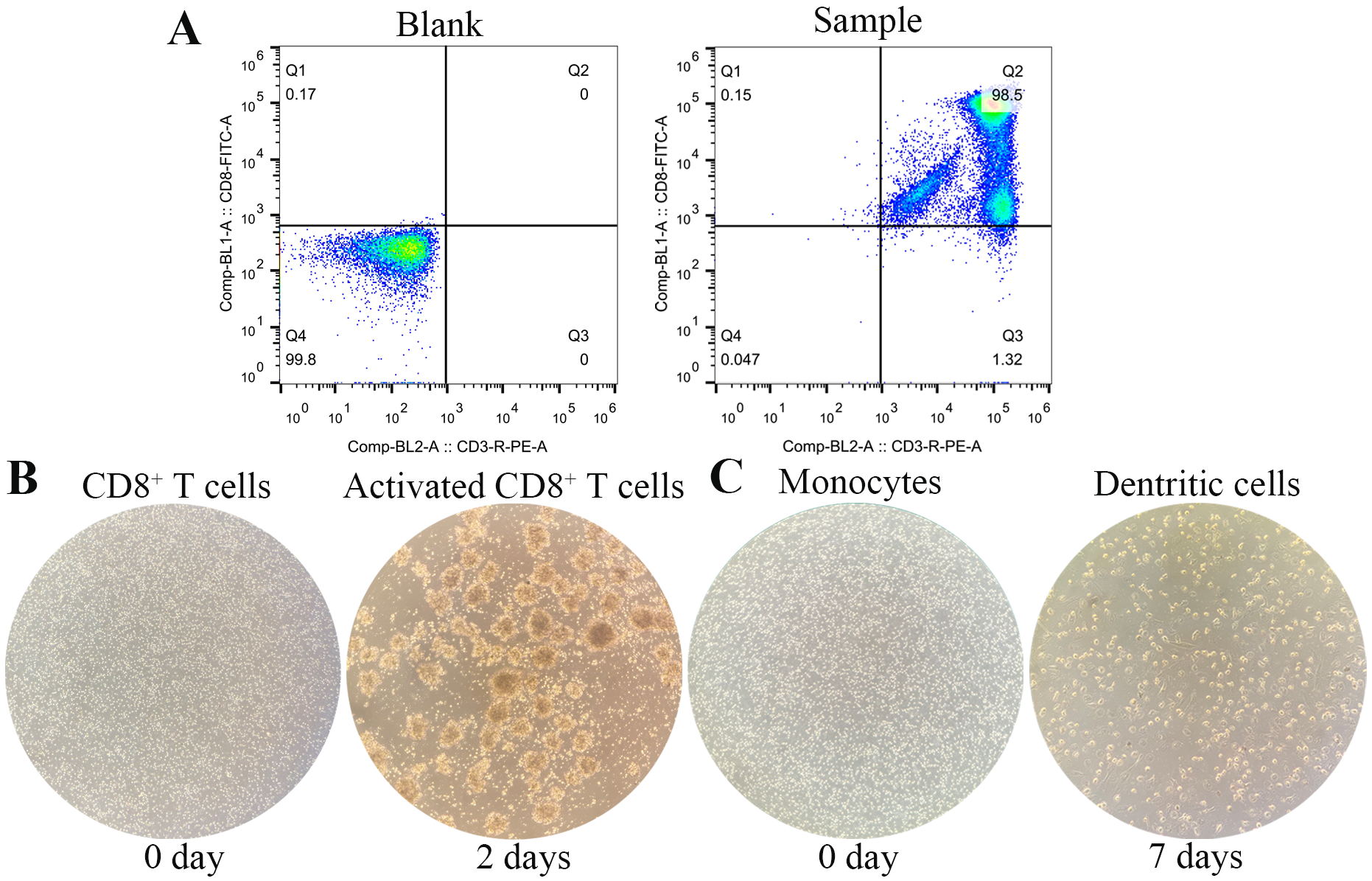

Supplement: Supplementary file 3 — Supplementary figure 1 [file 41419_2026_8743_MOESM3_ESM.tif]

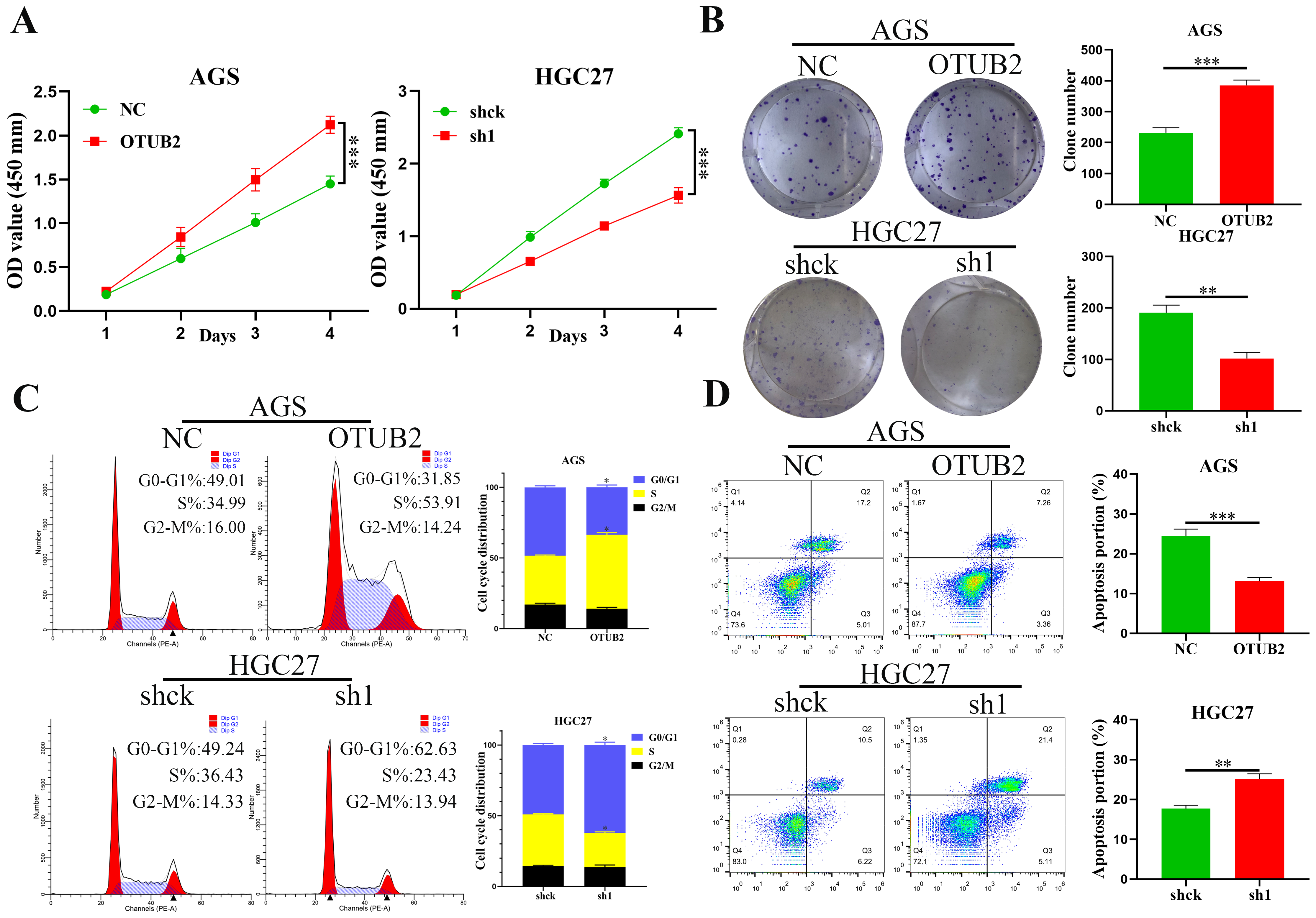

Supplement: Supplementary file 4 — Supplementary figure 2 [file 41419_2026_8743_MOESM4_ESM.tif]

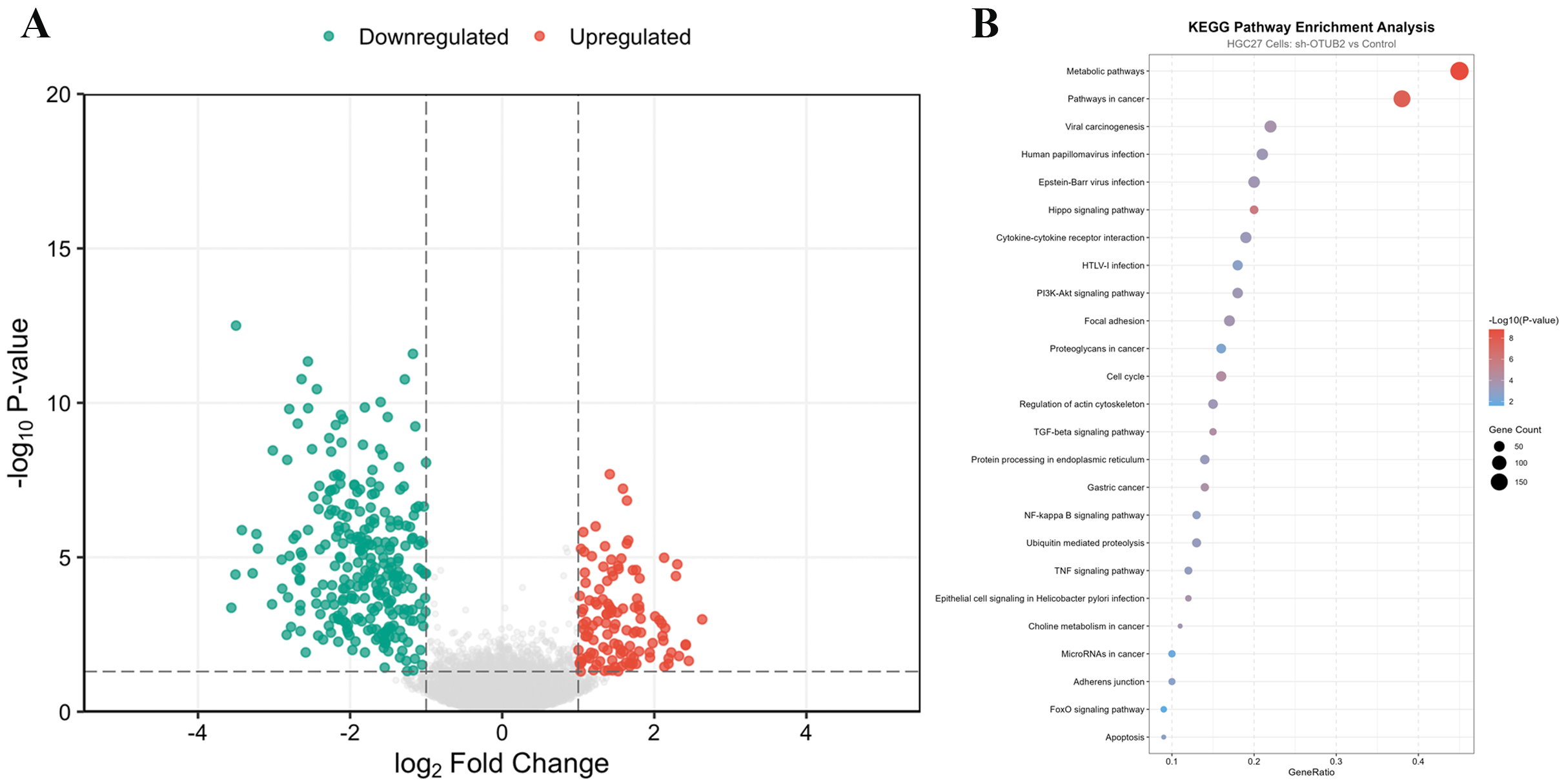

Supplement: Supplementary file 5 — Supplementary figure 3 [file 41419_2026_8743_MOESM5_ESM.tif]

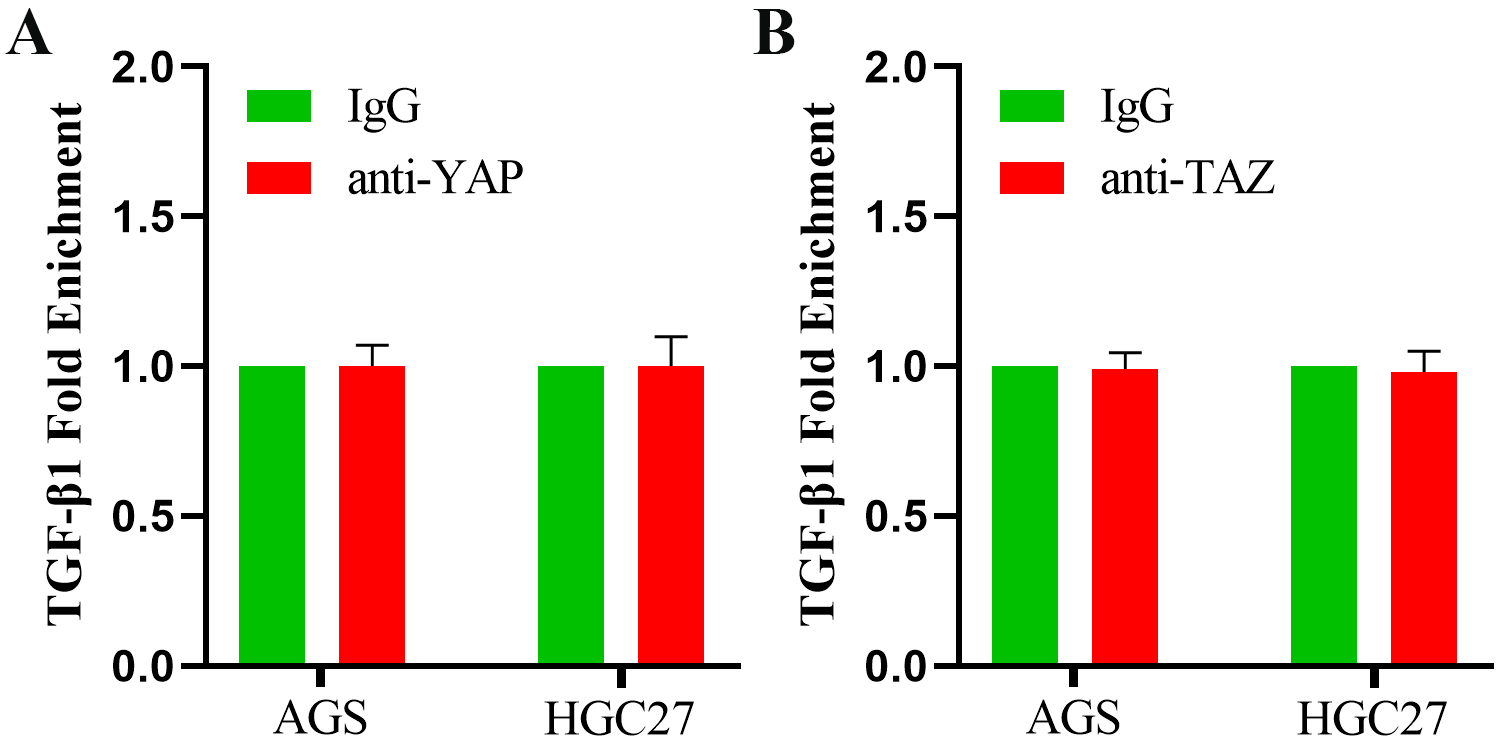

Supplement: Supplementary file 6 — Supplementary figure 3 [file 41419_2026_8743_MOESM6_ESM.tif]

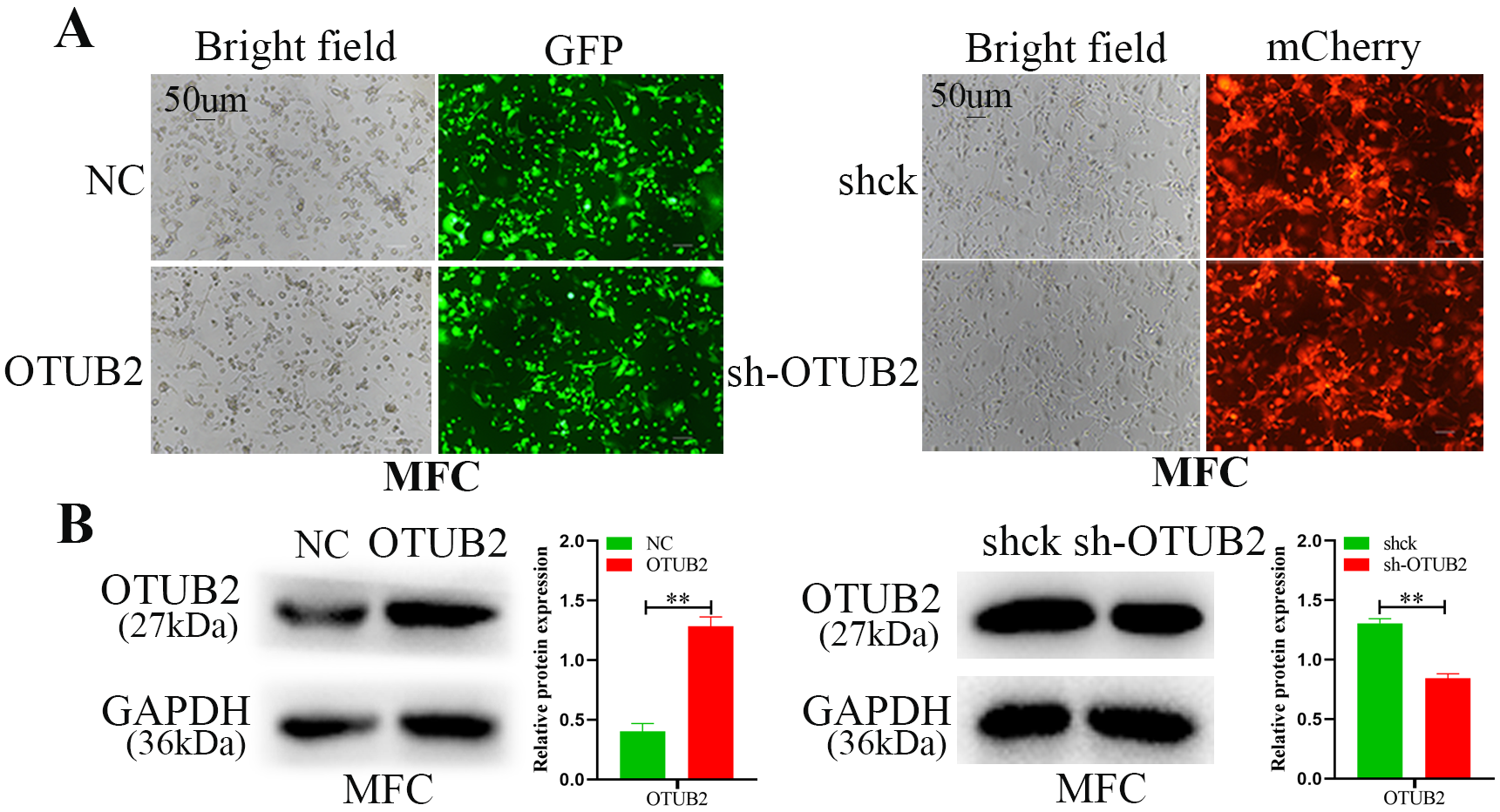

Supplement: Supplementary file 7 — Supplementary figure 5 [file 41419_2026_8743_MOESM7_ESM.tif]

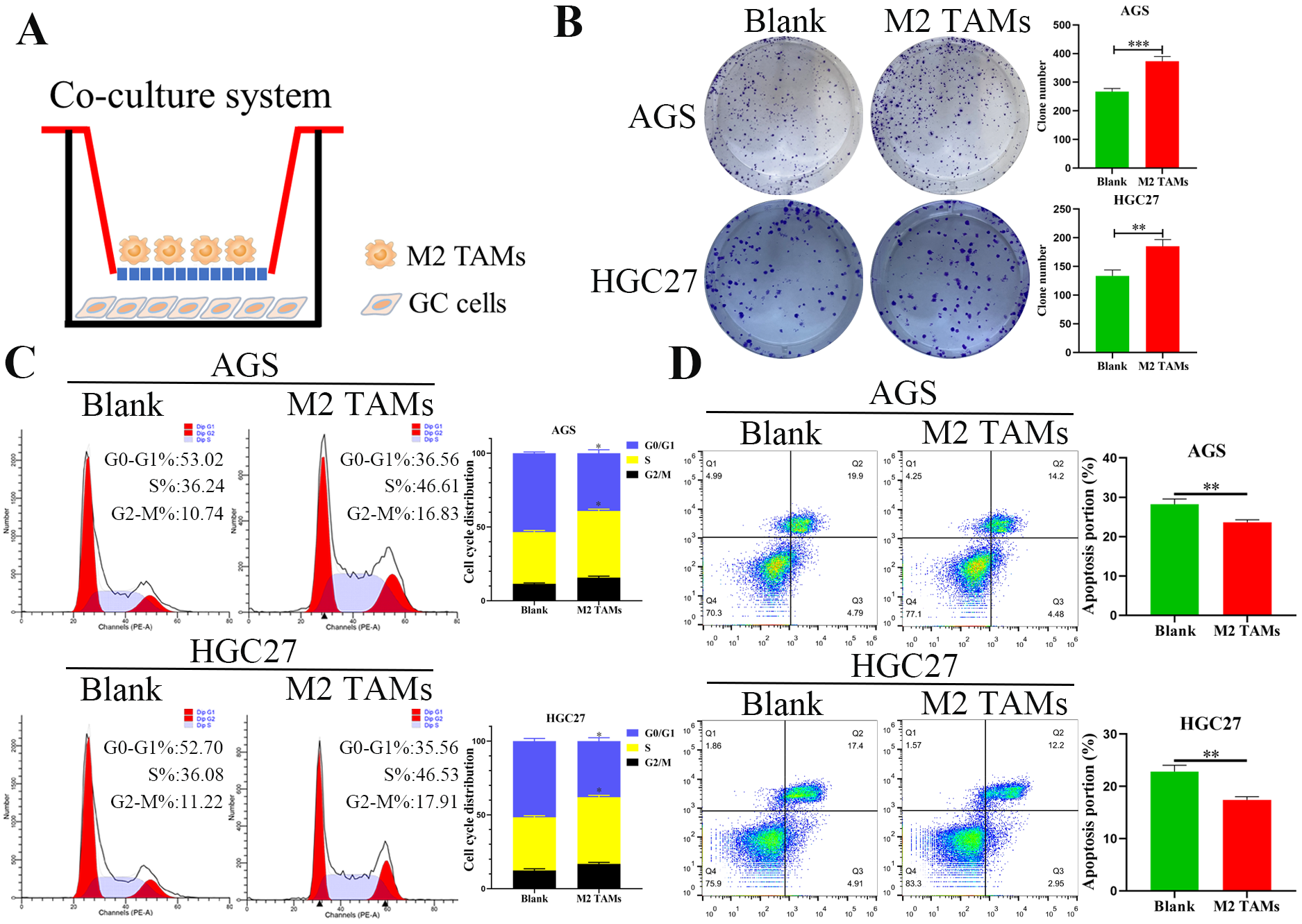

Supplement: Supplementary file 8 — Supplementary figure 6 [file 41419_2026_8743_MOESM8_ESM.tif]
