# Supplementary material for: OTUB2 induces M2 tumor-associated macrophage polarization and increases CD274 expression in gastric cancer cells to aggravate the progression of gastric cancer
Source: Cell Death Dis. 2026 Apr 15;17(1):509. doi: 10.1038/s41419-026-08743-9 (PMC13201613; doi:10.1038/s41419-026-08743-9)
Supplement: Supplementary file 9 — Supplementary table 1 [file 41419_2026_8743_MOESM9_ESM.docx]

**Table S1**. The shRNA sequences of OTUB2.

| ID | 5’ | stem | loop | stem | 3’ |
| --- | --- | --- | --- | --- | --- |
| OTUB2-RNAi(85340-1)-a | Ccgg | caTCCCACTACAACATCCTTT | CTCGAG | AAAGGATGTTGTAGTGGGATG | TTTTTg |
| OTUB2-RNAi(85340-1)-b | GATCCAAAAA | caTCCCACTACAACATCCTTT | CTCGAG | AAAGGATGTTGTAGTGGGATG |  |
| OTUB2-RNAi(85341-1)-a | Ccgg | cgAGATGGATACCGCCCTGAA | CTCGAG | TTCAGGGCGGTATCCATCTCG | TTTTTg |
| OTUB2-RNAi(85341-1)-b | GATCCAAAAA | cgAGATGGATACCGCCCTGAA | CTCGAG | TTCAGGGCGGTATCCATCTCG |  |
| OTUB2-RNAi(85342-1)-a | Ccgg | tgTGGTGGAACTGGTAGAGAA | CTCGAG | TTCTCTACCAGTTCCACCACA | TTTTTg |
| OTUB2-RNAi(85342-1)-b | GATCCAAAAA | tgTGGTGGAACTGGTAGAGAA | CTCGAG | TTCTCTACCAGTTCCACCACA |  |
